# Structure of the *Macrobrachium rosenbergii* nodavirus: A new genus within the *Nodaviridae*?

**DOI:** 10.1371/journal.pbio.3000038

**Published:** 2018-10-22

**Authors:** Kok Lian Ho, Mads Gabrielsen, Poay Ling Beh, Chare Li Kueh, Qiu Xian Thong, James Streetley, Wen Siang Tan, David Bhella

**Affiliations:** 1 Department of Pathology, Faculty of Medicine and Health Sciences, Universiti Putra Malaysia, UPM Serdang, Selangor, Malaysia; 2 CRUK Beatson Institute, Garscube Campus, Glasgow, Scotland United Kingdom; 3 Department of Microbiology, Faculty of Biotechnology and Biomolecular Sciences, Universiti Putra Malaysia, UPM Serdang, Selangor, Malaysia; 4 MRC-University of Glasgow Centre for Virus Research, Garscube Campus, Glasgow, Scotland, United Kingdom; 5 Institute of Bioscience, Universiti Putra Malaysia, UPM Serdang, Selangor Malaysia; University of Pittsburgh, UNITED STATES

## Abstract

*Macrobrachium rosenbergii* nodavirus (*Mr*NV) is a pathogen of freshwater prawns that poses a threat to food security and causes significant economic losses in the aquaculture industries of many developing nations. A detailed understanding of the *Mr*NV virion structure will inform the development of strategies to control outbreaks. The *Mr*NV capsid has also been engineered to display heterologous antigens, and thus knowledge of its atomic resolution structure will benefit efforts to develop tools based on this platform. Here, we present an atomic-resolution model of the *Mr*NV capsid protein (CP), calculated by cryogenic electron microscopy (cryoEM) of *Mr*NV virus-like particles (VLPs) produced in insect cells, and three-dimensional (3D) image reconstruction at 3.3 Å resolution. CryoEM of *Mr*NV virions purified from infected freshwater prawn post-larvae yielded a 6.6 Å resolution structure, confirming the biological relevance of the VLP structure. Our data revealed that unlike other known nodavirus structures, which have been shown to assemble capsids having trimeric spikes, *Mr*NV assembles a T = 3 capsid with dimeric spikes. We also found a number of surprising similarities between the *Mr*NV capsid structure and that of the Tombusviridae: 1) an extensive network of N-terminal arms (NTAs) lines the capsid interior, forming long-range interactions to lace together asymmetric units; 2) the capsid shell is stabilised by 3 pairs of Ca^2+^ ions in each asymmetric unit; 3) the protruding spike domain exhibits a very similar fold to that seen in the spikes of the tombusviruses. These structural similarities raise questions concerning the taxonomic classification of *Mr*NV.

## Abstract 2

Macrobrachium rosenbergii nodavirus (MrNV) is an economically important pathogen of freshwater prawns. Determination of the structure of the MrNV virion by cryo-electron microscopy reveals a capsid protein that is highly divergent from other known nodaviruses but has a surprising resemblance to plant-infecting tombusviruses.

## Author summary

The freshwater prawn *Macrobrachium rosenbergii* is widely cultivated for food. Production is threatened by *Macrobrachium rosenbergii* nodavirus (*Mr*NV), the causative agent of white-tail disease. Outbreaks in hatcheries often result in mortality rates of up to 100% in larvae and post-larvae, leading to devastating economic losses and threatening food security. We describe the atomic structure of the *Mr*NV capsid, solved by cryogenic electron microscopy and three-dimensional image reconstruction. Our analysis revealed surprising differences between the structure of *Mr*NV and that of other known nodaviruses. Moreover, we observed several features in the *Mr*NV capsid that have been previously described in virion structures of the plant-infecting tombusvirus family. Most notably, the *Mr*NV capsid exhibits pronounced dimeric spikes on its surface, the topology of this region closely resembling tombusvirus capsid spikes. Known nodavirus structures have trimeric spikes and do not display the same protein fold. The *Mr*NV capsid is stabilised by divalent cations and laced together by a network of N-terminal arms that line the interior of the virion. Our analysis raises questions about the taxonomic classification of *Mr*NV as well as revealing the structure of the capsid of this important pathogen. These data have the potential to inform the development of future interventions to prevent white-tail disease.

## Introduction

The giant freshwater prawn *M*. *rosenbergii* is widely cultivated in tropical and subtropical areas for food. The global production of this species has increased dramatically from about 3,000 tons in 1980 to more than 220,000 tons in 2014 [1]. However, productivity is threatened by white-tail disease (WTD), which is caused by *M*. *rosenbergii* nodavirus (*Mr*NV). This often leads to 100% mortality rates in larvae and post-larvae of *M*. *rosenbergii* [2]. The first *Mr*NV outbreak was reported in Pointe Noire, Guadeloupe in 1997, followed by China [3], India [4], Taiwan [5], Thailand [6], Malaysia [7], Australia [8], and recently Indonesia [9]. To date, neither a vaccine nor effective treatment is available to prevent or manage *Mr*NV outbreaks.

*Mr*NV has been classified within the Nodaviridae family of viruses. These nonenveloped viruses have bipartite, positive-sense, single-stranded RNA genomes that are packaged within T = 3 icosahedral capsids. Presently, this family has 2 established genera: *Alphanodavirus* and *Betanodavirus*. The former consists of insect-infecting nodaviruses such as Flock House virus (FHV), Pariacoto virus (PaV), black beetle virus (BBV), Nodamura virus (NoV), and Boolarra virus (BoV), while the latter contains fish-infecting nodaviruses such as Malabaricus grouper nervous necrosis virus (MGNNV), grouper nervous necrosis virus (GNNV), and striped jack nervous necrosis virus (SJNNV). Although *Mr*NV is classified within the Nodaviridae, amino acid sequence comparison revealed that its capsid protein (CP) shares low similarity (less than 20%) with other nodaviruses in the 2 established genera. Thus, it is ambiguous whether *Mr*NV should be grouped in either one of these genera. Conversely, the amino acid sequence of *Mr*NV CP shares approximately 80% similarity with that of *Penaeus vannamei* nodavirus (*Pv*NV). These 2 crustacean nodaviruses have therefore been proposed to be grouped into a new genus: *Gammanodavirus* [10].

Nodaviruses are very simple, with their 2 short-genomic RNAs encoding 3 gene products. RNA 1 (3.2 kb) encodes the RNA-dependent RNA polymerase (RdRp) and the nonstructural B2-like protein, while RNA 2 (1.2 kb) encodes the viral CP. The full-length *Mr*NV CP is a polypeptide of 371 amino acids. The N-terminal arginine-rich region interacts with the RNA genome [11], while the C-terminal domain plays crucial roles in host cell attachment and internalisation [12]. A nuclear localisation signal (NLS) has also been identified at the N-terminus (N-ter) (amino acids 20–29) of CP. This has been shown to target the viral capsid to the nucleus of insect cells [13]. Further functional regions of CP have yet to be defined.

Several three-dimensional (3D) structures of alpha- and betanodaviruses have been determined using both X-ray crystallography and cryogenic electron microscopy (cryoEM) [14–18]. These analyses have revealed several common features. Nodaviruses assemble T = 3 icosahedral capsids; 180 CP protomers assemble such that the asymmetric unit comprises 3 identical capsid subunits in 3 quasiequivalent positions termed A, B, and C (here termed CP_A_, CP_B_, and CP_C_). To date, all alpha- and betanodaviruses have been found to have capsomeres that present a trimeric spike. The arginine-rich N-terminal region of the viral CP interacts with the viral RNA segments (exemplified by PaV, Protein Data Bank [PDB]: 1F8V [15]), leading to the formation of a dodecahedral RNA cage at the virion interior.

We have previously shown that recombinant CP of *Mr*NV produced using baculovirus expression in *Spodoptera frugiperda* (Sf9) cells assembles into virus-like particles (VLPs) with a diameter of approximately 40 nm [19]. We determined the intermediate resolution structure of the *Mr*NV capsid using cryoEM and image reconstruction [20]. At this resolution, our reconstruction revealed distinct dimer-clustering of capsomeres in the T = 3 *Mr*NV icosahedral capsid. Capsomeres were seen to form square, thin, and blade-like spikes on the virion surface. All other nodaviruses have been shown to assemble with trimeric capsomers. Our structure therefore revealed a strikingly divergent morphology for *Mr*NV, lending weight to the proposed classification of *Mr*NV within a new genus of nodaviruses [20].

Here, we present a high-resolution 3D reconstruction of the *Mr*NV VLP, solved at 3.3 Å resolution. From these data, we have constructed an atomic model of the *Mr*NV CP. We show that the shell (S) domain of *Mr*NV CP possesses the canonical 8-stranded β-barrel structure common to all nodaviruses. There are, however, striking structural similarities between the *Mr*NV capsid and those of members of the Tombusviridae. The protruding (P) domain exhibits a similar fold to that which has been previously shown for tomato bushy stunt virus (TBSV), although the spikes are narrower and are oriented quite differently in the 2 dimeric forms (AB and CC). CP–CP interactions in the S domain are stabilised by coordinated Ca^2+^ ions. Protomers forming CC dimers, located at the icosahedral 2-fold symmetry axes of the capsid, possess an ordered N-terminal arm (NTA). This passes along the capsid interior, forming intermolecular interactions with neighbouring protomers to stabilise the capsid. Unlike the NTA of TBSV, however, which folds back to form an additional β-strand in CP_C_ before going on to form a structure known as a β-annulus at the adjacent icosahedral 3-fold axis, the NTA of the *Mr*NV CP_C_ crosses the icosahedral 2-fold symmetry axis and inserts into a symmetry-related CP_B_. The NTA then forms a β-annulus at the next nearest 3-fold symmetry axis before continuing to pass along the capsid interior, donating a strand to a β-sheet in a second neighbouring CP_B_ molecule.

We also present an intermediate-resolution structure of the authentic *Mr*NV virion, purified from infected larvae of *M*. *rosenbergii*, suggesting that the infectious virus exhibits an identical capsid structure to the one that we have determined for the VLP. Our data present a detailed structural view of this economically important pathogen and raise questions concerning the taxonomic classification of both *Mr*NV and the related *Pv*NV.

## Results

### CryoEM of *Mr*NV VLPs

**Fig 1:**
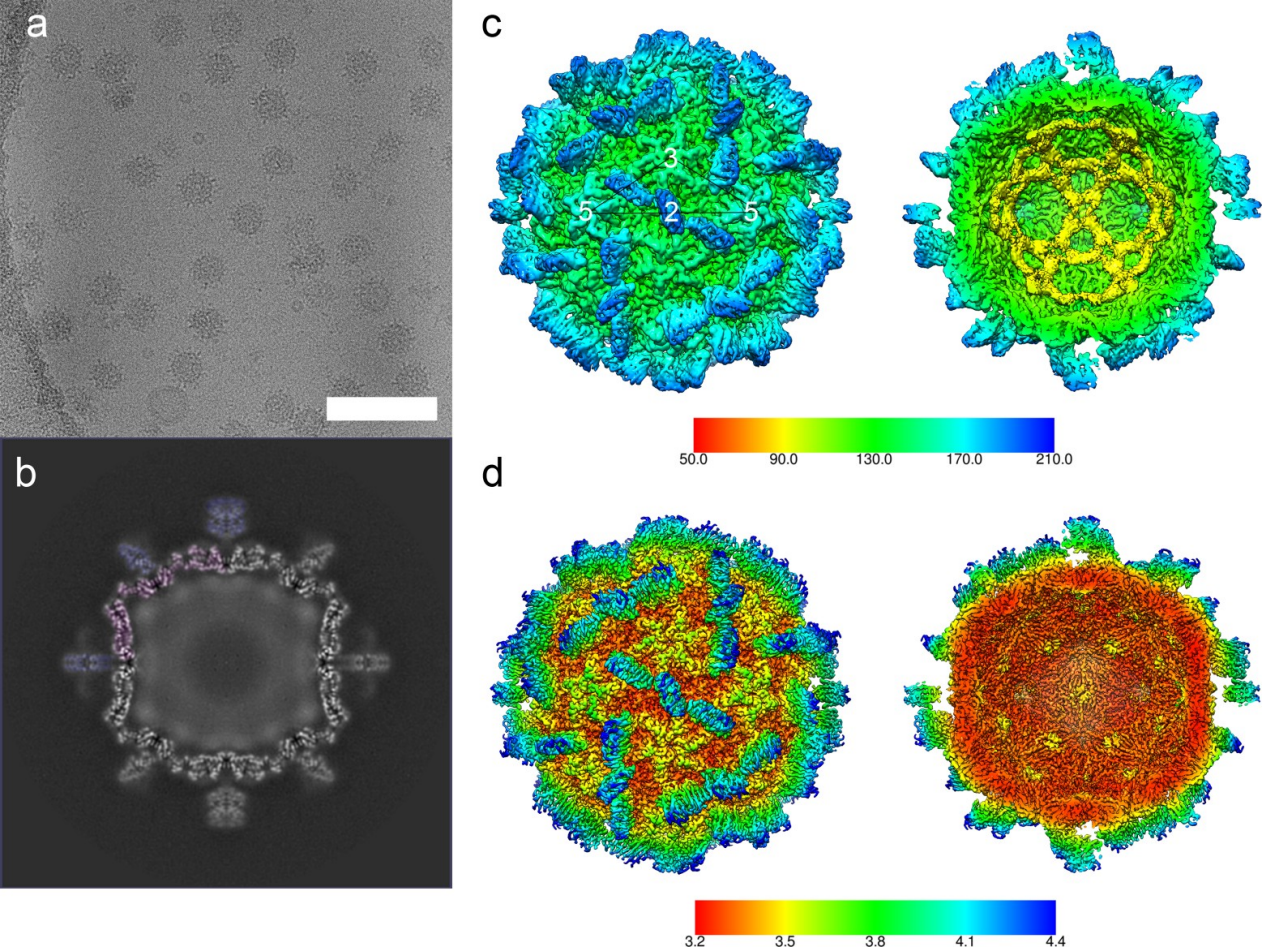
CryoEM and 3D reconstruction of *Mr*NV VLPs. CryoEM of *Mr*NV VLPs revealed particles that were 40 nm in diameter with pronounced spikes on their outer surface (a, scale bar = 100 nm). A central section through the icosahedral reconstruction of the *Mr*NV VLP reveals a sharply defined capsid shell (partially tinted pink) containing fuzzy density that we attribute to packaged nucleic acids. The P-domain spikes (partially tinted blue) were less well resolved in comparison to the shell, most likely a consequence of flexibility (b). An isosurface view of the reconstruction is shown coloured by radius and viewed along the icosahedral 2-fold symmetry axis (the symmetry axes are indicated by white numbers). A cutaway view reveals that the internal density forms a dodecahedral cage, consistent with that described for other nodaviruses (c). The sharpened map is also presented, coloured according to resolution, as both external and cutaway views (d). Sharpening of cryoEM maps down-weights lower-resolution information to reveal the fine structural details of the map, such as amino acid side chains. Poorly defined features such as the packaged nucleic acids are often lost upon sharpening because no high-resolution information is present. CryoEM, cryogenic electron microscopy; *Mr*NV, *M*. *rosenbergii* nodavirus; P domain, protruding domain; 3D, three-dimensional; VLP, virus-like particle.

To calculate an atomic model of the *Mr*NV CP, we sought to determine a high-resolution 3D reconstruction of the capsid. Frozen hydrated preparations of *Mr*NV VLPs were imaged in a Thermo-Fisher Titan Krios at the United Kingdom Electron Bio-Imaging Centre (eBIC) (Fig 1A). A total of 40,883 particle images were used to calculate a reconstruction with an overall resolution of 3.3 Å (Fig 1B and 1C and S1 Fig, S1 Movie). The reconstructed density map closely matched our previously published 7 Å structure of the *Mr*NV VLP produced in *S*f9 cells, showing pronounced blade-shaped dimeric spikes on the capsid exterior and a dodecahedral cage of RNA density within the particle. A cross section through the reconstructed density revealed that the S domains of the VLP were sharply resolved, while the P domains were less well defined, having weaker, fuzzier density (Fig 1B). Local resolution analysis confirmed this, revealing that much of the S domain was solved to 3.2 Å resolution, while the tips of the dimeric capsomeres were poorer than 4 Å resolution (Fig 1D). Local resolution filtering and sharpening was applied with a B-factor of −140 Å^2^ to generate a density map that was suited for high-resolution model building.

### An atomic model of the *Mr*NV VLP asymmetric unit

The asymmetric unit of the T = 3 *Mr*NV capsid comprises 3 copies of *Mr*NV CP; CP_A_, CP_B_, and CP_C_. We have previously shown that the P domains assemble to form dimeric spikes, with AB dimers arranged about the 5-fold symmetry axes and the CC dimers located at the 2-fold symmetry axes. We set out to build the sequence of the *Mr*NV CP (S2 Fig) into our density map to produce an atomic model of the *Mr*NV CP for each quasiequivalent position. As a starting point, we docked a homology model into our density map [12, 20]. Overall, this model fitted poorly within the reconstructed density map, with the exception of 2 regions between amino acid residues 104–135 and 232–243. A model for the S domain of CP_A_ was therefore manually built and refined from this starting point. This partial model was then docked to CP_B_ and CP_C_ and further edited and refined, leading to reliable models for the S domains at each quasiequivalent position. Interestingly, our density map presented density consistent with the presence of 2 metal ions per CP. Based on the surrounding residues and distances to coordinating atoms, we have modelled these as calcium ions (discussed below). In our 3D reconstruction, density for the S domain was very well resolved. This allowed us to model this region to a high degree of confidence and with relative ease.

**Fig 2:**
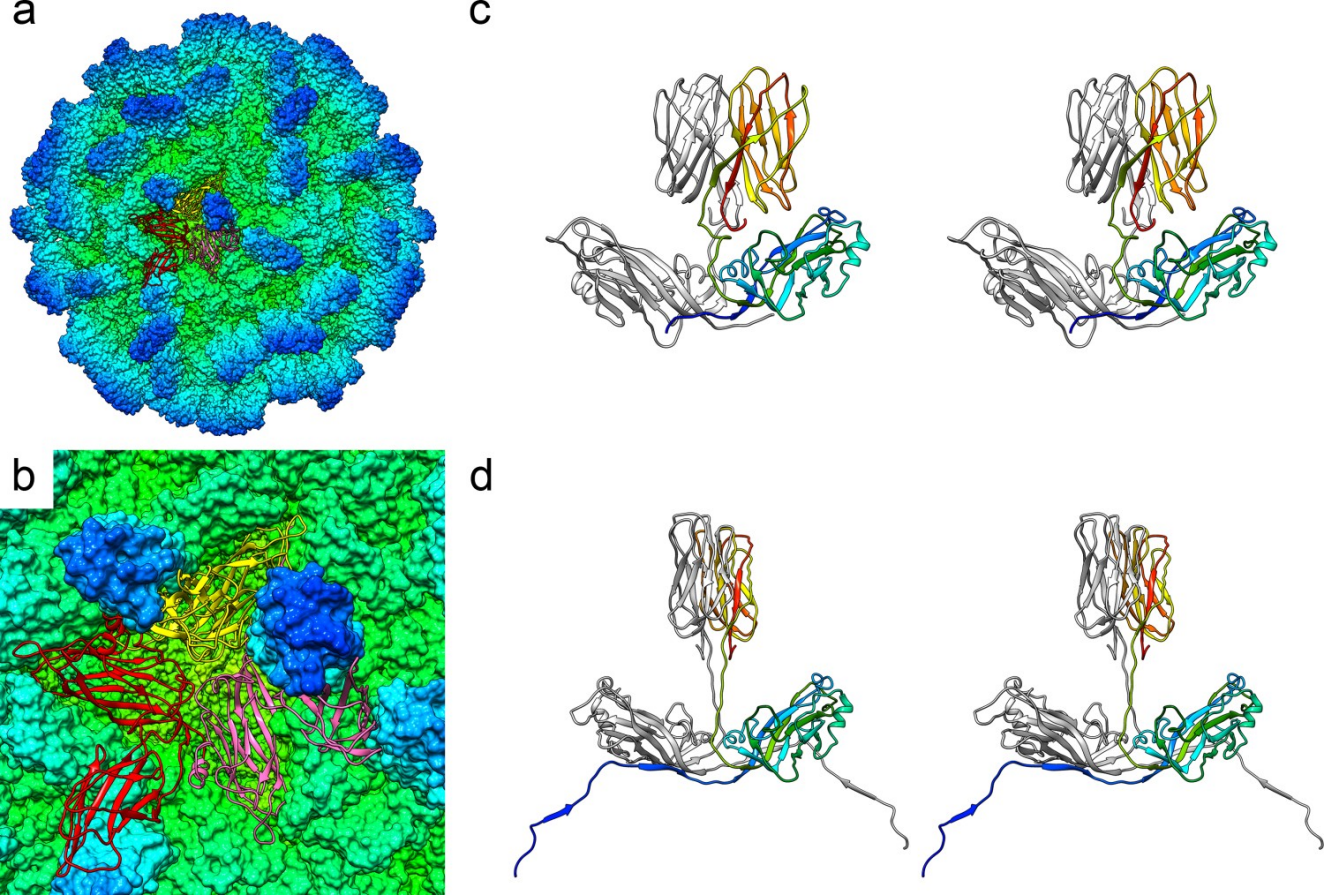
Atomic model of the *Mr*NV capsid. A solvent-excluded surface of the entire capsid is shown, coloured by radius (see Fig 1C for key). A single asymmetric unit comprising 3 copies of CP—CP_A_ (red), CP_B_ (yellow), and CP_C_ (pink)—is shown as a ribbon diagram (a) and close-up view (b). Wall-eyed stereo-paired views of each dimer are shown (AB dimer [c], CC dimer [d]). In (c), CP_B_ is presented in rainbow colour scheme, while CP_A_ is coloured grey. In (d), one CP_C_ chain is shown in rainbow colour scheme, while the other is grey. CP, capsid protein; *Mr*NV, *M*. *rosenbergii* nodavirus.

The P domains were, however, rather less well defined and presented a more challenging task, particularly at the distal tips of the dimeric spikes. CP_B_ was found to be the best resolved as judged by continuity of density, while CP_C_ appeared the least well resolved. Throughout the amino acid sequence of the P domain, there are bulky amino acid side chains that gave confidence in our interpretation of the map. Following several rounds of manual editing and refinement, a model was achieved for the full asymmetric unit that matched our density map and had reasonable geometry (Fig 2, S2 Movie, S1 Table).

### A β-annulus motif formed of multiple NTAs of chain C

**Fig 3:**
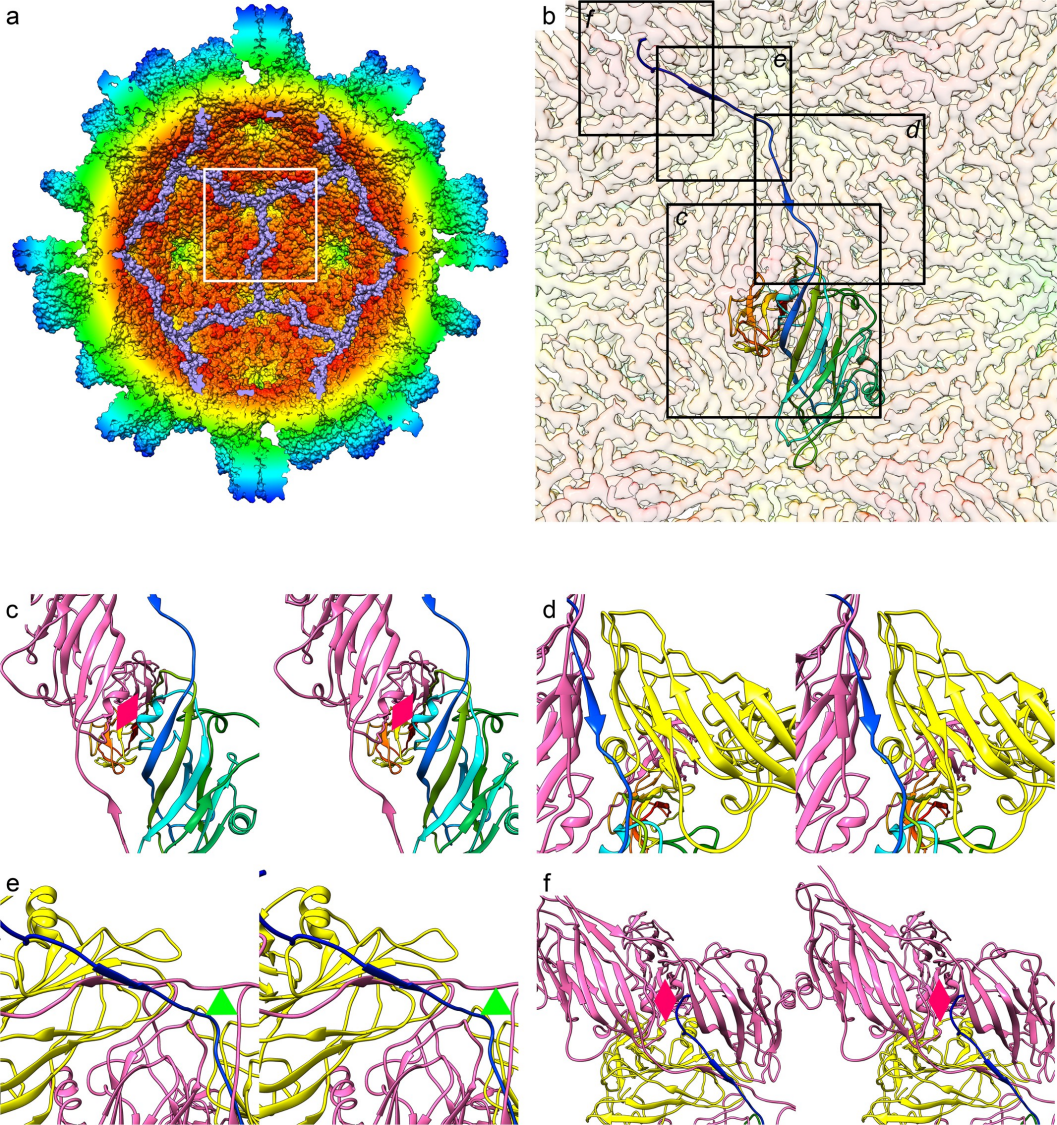
The CP_C_ NTA forms an extensive network at the capsid interior that includes a β-annulus motif. A solvent-excluded surface view of the *Mr*NV capsid is shown, coloured by radius and clipped to reveal the capsid interior. The CP_C_ NTAs are coloured mauve to highlight the extended network formed (a). The white frame highlights the view presented in (b), in which the cryoEM map is shown as a transparent isosurface with a single CP_C_ molecule presented as a ribbon diagram with rainbow colouring. Black frames highlight the views presented in subsequent figures (c–f). (c) A wall-eyed stereo-pair view of a CC dimer from the capsid interior shows the NTA emerging from the S-domain β-jelly roll to cross the icosahedral 2-fold symmetry axis (pink diamond). (d) Moving from C- to N-termini, the NTA runs between the symmetry-related CP_C_ (pink) and an adjacent CP_B_ (yellow), donating a strand to a β-sheet within the CP_B_. This sheet also includes a β-strand donated by a symmetry-related CP_C_ NTA (pink). (e) The NTA then forms a β-annulus at the icosahedral 3-fold axis (green triangle) and donates a strand to a β-sheet within a second CP_B_ (yellow), which similarly also includes a β-strand from a symmetry related CP_C_ (pink). Thus, CP_C_ NTAs are interdigitated between other CP_C_ NTAs and CP_B_ S domains. (f) The N-terminal 29 residues of CP_C_ are not resolved in our cryoEM map; however, the last visible CP_C_ NTA density terminates beneath a symmetry-related icosahedral 2-fold symmetry axis (pink diamond), suggesting that the RNA binding site is proximal to this region. CP, capsid protein; cryoEM, cryogenic electron microscopy; *Mr*NV, *M*. *rosenbergii* nodavirus; NTA, N-terminal arm; P domain, protruding domain; S domain, shell domain.

The N-terminal regions of CP that include the RNA binding sites (amino acid residues 21–29) were not resolved for any of the chains (CP_A_, CP_B_, or CP_C_) in our density map. For CP_A_, we have successfully modelled amino acid residues 56–371, while for CP_B_, we were able to build amino acid residues 55–371. CP_C_ has a well-resolved NTA that allowed modelling from amino acid 31. Interestingly, the CP_C_ NTA was found to form extensive contacts with symmetry-related CP_B_ and CP_C_ molecules, forming a network that crosses the capsid interior and is reminiscent of the NTAs previously described for several tombusviruses (Fig 3, S3 Movie). The CC dimer interface lies at the icosahedral 2-fold axis. Each CP_C_ NTA emerges from the S domain close to this symmetry axis and interacts with 2 CP_B_ protomers, donating β-strands to β-sheets within the CP_B_ S domains. The CP_C_ NTA extends across the CC dimer (and icosahedral) 2-fold symmetry axis and inserts into the first CP_B_ subunit, which lies adjacent to the symmetry-related CP_C_ subunit (Fig 3C and 3D). Moving from the C-terminus (C-ter) to the N-ter, the NTA then crosses the adjacent icosahedral 3-fold axis and inserts into the next nearest CP_B_ subunit, donating a second β-strand to the β-sheet comprising that molecule and a symmetry-related CP_C_ NTA (Fig 3E and 3F). This interdigitated arrangement of NTAs extending from CPs at the C-position was first described for TBSV [21] and termed a β-annulus owing to the manner in which CP_C_ NTAs wrap around each other at the icosahedral 3-fold axes. Nevertheless, in the case of *Mr*NV, the lacing together of CP_C_ molecules is more extensive because the NTA does not fold back on the CP_C_ to emerge from the S domain at the nearest 3-fold axis and there form the β-annulus structure (as it does in TBSV). Rather, it crosses the 2-fold axis of the CC dimer and then inserts into 2 CP_B_ molecules arranged about the opposite 3-fold axis, where the β-annulus is formed. The last resolved N-terminal residue (Pro31) lies under the next neighbouring 2-fold axis. This is related to the originating 2-fold by a counterclockwise rotation of 120^o^ about the 3-fold axis of the β-annulus (viewed from the capsid exterior). Although it is not resolved, the arginine-rich putative RNA binding site (amino acids 21–29) must therefore be located proximal to the 2-fold symmetry axes for the CP_C_ chains.

### Coordinated metal ions stabilise CP–CP interactions in the S domain

**Fig 4:**
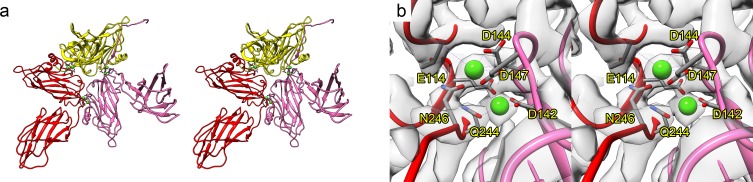
Metal ions stabilise the *Mr*NV asymmetric unit. Within our cryoEM map, we saw density consistent with the presence of coordinated metal ions. A wall-eyed stereo-paired view of a ribbon diagram of the asymmetric unit is shown with chains coloured A: red, B: yellow, and C: pink. Green spheres highlight the positions of the putative metal ions, which we have modelled as Ca^2+^ (a). A close-up view of a pair of metal ions at the interface between chains A and C is also shown in stereo (b). The amino acid residues that make up the coordination spheres are labelled. The cryoEM density map is also shown as a transparent isosurface. CryoEM, cryogenic electron microscopy; *Mr*NV, *M*. *rosenbergii* nodavirus.

The *Mr*NV CP S domain comprises residues 62–242 and forms the contiguous shell of the capsid. The T = 3 assembly is made up of 180 copies of the canonical 8-stranded antiparallel β-barrel fold, known as the β-jelly roll. This is commonly seen in positive-sense RNA-containing viruses, including both the nodaviruses and tombusviruses. Another interesting parallel between the structure of *Mr*NV and the tombusviruses is the presence of coordinated metal ions at the interface between CP subunits (Fig 4, S4 Movie). X-ray crystallographic difference mapping of TBSV following EDTA treatment identified 2 divalent-cation–binding sites at the interface between CPs within the asymmetric unit, which were modelled as Ca^2+^ [22]. Chelation followed by a rise in pH (>7.0) has been shown to cause a structural transition to a ‘swollen’ state in these virions, indicating that these divalent cations play a role in virion stabilisation or possibly control of uncoating. Based on the striking similarity in the locations of these putative metal ions in our data compared to those previously published for TBSV (PDB: 2TBV), and the surrounding residues, we have modelled these putative metal ions as calcium (Fig 4B).

### A flexible hinge leads to differently oriented P-domain dimer spikes

We have previously noted the striking differences in the orientations of the P-domain dimer spikes, relative to the underlying capsid shell, between AB and CC dimers. The CC-dimer spike is rotated approximately 85° counterclockwise relative to that of the AB dimer (viewed from the capsid exterior). Moreover, CC-dimer P domains are raised from the capsid surface upon legs of density, whereas the AB P domains sit closer to the capsid shell and are tilted towards their nearest 2-fold symmetry axis. Our atomic model of the *Mr*NV VLP reveals the reason for the substantial differences in pose of these 2 capsomere forms. There is a large linker region between the S and P domains at amino acid residues 241–258. In the CC-dimer, this linker emerges from the S-domain β-jelly roll and forms a straight leg that is normal to the capsid surface (Fig 2E). The interdomain linker in CP_A_ and CP_B_, on the other hand, has 2 bends: one between residues Pro247 and Pro249, which causes the linker to make a right-angled turn, and another at Ile252–Gln254, which likewise causes a right-angled turn, restoring the path of the linker to its original radial orientation (Fig 2C). The twist in the linkers at the AB dimer induced by these turns therefore accounts for the major differences in the orientations of the 2 types of spike. Interestingly, although we previously noted that the CP_B_ P domain was more closely apposed to the S domain than CP_A_, our model does not show any contacts. The AB P domain’s orientation is defined by interactions within the AB linker region and with the CP_C_ P domain (S5 Movie).

### Intercapsomere contacts in the P domain lead to the formation of a blade-like superstructure

**Fig 5:**
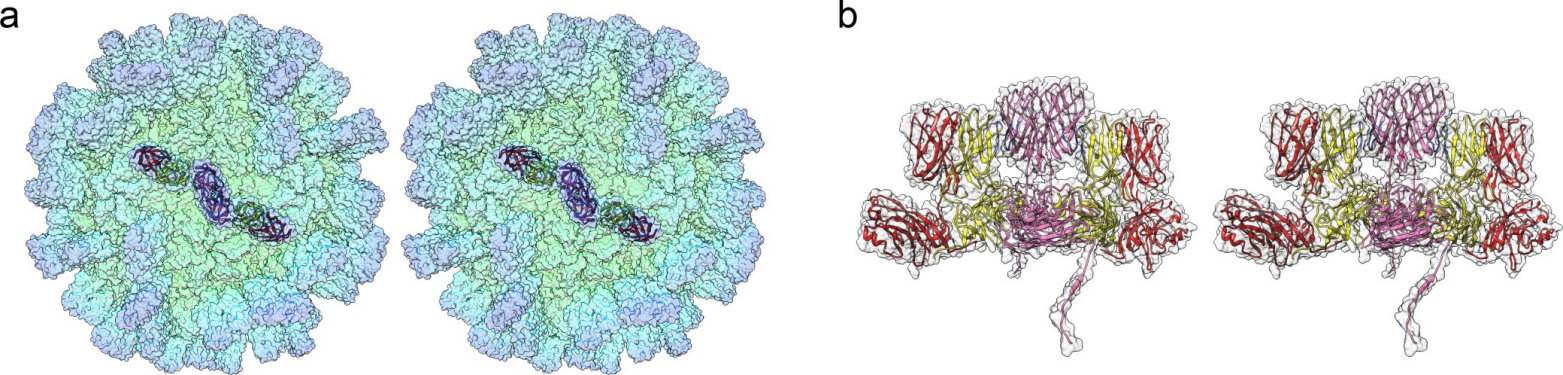
A superstructure comprising the P domains of 2 AB dimers and 1 CC dimer. A transparent solvent-excluded surface of the *Mr*NV capsid is presented as a stereo-paired view (a). Six P domains are shown as ribbon diagrams to highlight the formation of a superstructure that lays across the icosahedral 2-fold symmetry axis. A side view of the superstructure shows how P-domain AB dimers act as buttresses to stabilise the CC-dimer spike. The contact residues in CP_B_ (yellow) and CP_C_ (pink) are highlighted in shades of blue. CP, capsid protein; *Mr*NV, *M*. *rosenbergii* nodavirus; P domain, protruding domain.

CC-dimer spikes are less well resolved in our cryoEM map than those of the AB dimers. This is to be expected given the manner in which the AB-dimer spike is stabilised through interactions in the AB linker. In contrast, the P domains of CP_C_ stand on extended polypeptide legs that may not offer the same support. The CC spike is instead stabilised by contacts between the P domains of CP_B_ and CP_C_. AB spikes act as buttresses to the CC capsomere through polar interactions between amino acid residues 270–276 of CP_B_ and 307–317 of CP_C_ (Fig 5, S5 Movie). This gives rise to the formation of a blade-like superstructure that lays across the 2-fold symmetry axis and comprises 1 CC dimer and 2 AB dimers.

### The *Mr*NV CP P-domain fold closely resembles that of the tombusviruses

**Fig 6:**
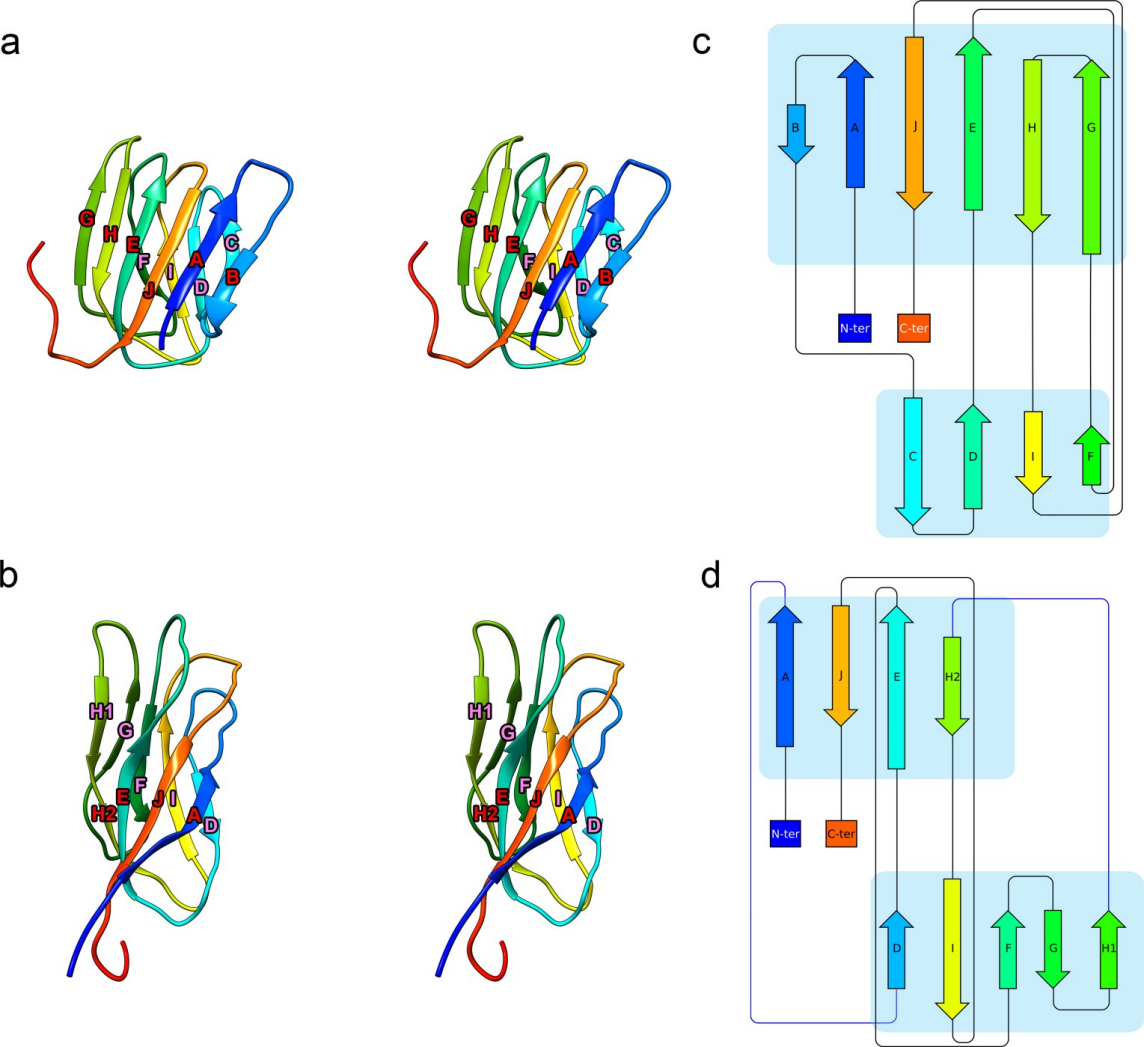
The *Mr*NV P domain has a similar fold to that of the tombusvirus CNV. A stereo-paired view of the CNV CP_B_ P domain (PDB: 4LLF) is presented as a ribbon diagram with rainbow colouring (a). The diagram is annotated to indicate successive β-strands from the N- to C-termini that together make up a 10-stranded antiparallel β-barrel. A similar motif is seen in the *Mr*NV P domain, which is likewise presented and annotated (b). Protein topology diagrams of CNV CP_B_ P domain (c) and *Mr*NV CP_B_ P domain (d) present a simplified view to highlight the similarities of the P-domain folds in these 2 viruses. C-ter, C-terminus; CNV, cucumber necrosis virus; CP, capsid protein; *Mr*NV, *M*. *rosenbergii* nodavirus; N-ter, N-terminus; P domain, protruding domain; PDB, Protein Data Bank.

It is noteworthy that the previously described homology model for the *Mr*NV CP structure [12] was based on the CP of a tombusvirus, cucumber necrosis virus (CNV; PDB: 4LLF [23]), rather than other known nodavirus structures. Although the homology model was a poor match for our cryoEM density map, our analysis has confirmed the hypothesised dimer-clustered T = 3 icosahedral capsid structure. Close inspection of the fold of the *Mr*NV P domain also reveals an unexpected structural homology between this nodavirus and the tombusviruses (Fig 6). Tombusvirus P domains have been shown to comprise a 10-stranded antiparallel β-barrel made up of 2 β-sheets annotated as BAJEHG and CDIF (Fig 6A and 6C). Secondary structure assignment in the P domains of the *Mr*NV structure was challenging owing to the poorer resolution in this region. Density in the P domain of CP_B_ was found to be the most clearly resolved, allowing us to build a model in which we have identified a similar β-barrel motif composed of 9 strands arranged into 2 β-sheets (S3 Fig). Based on a 3D pairwise alignment of the P domains for CP_B_ of CNV and *Mr*NV, we have annotated this fold as AJEH2 and DIFGH1 (Fig 6B and 6D, S4 Fig).

### Authentic *Mr*NV virions assemble capsids indistinguishable from VLPs

**Fig 7:**
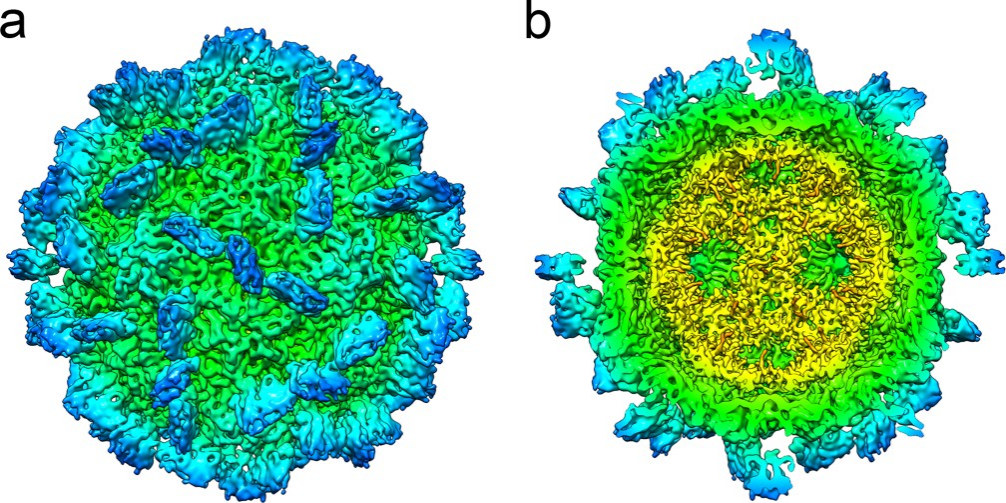
Icosahedral reconstruction of the authentic *Mr*NV virion at 6.6 Å resolution. CryoEM was used to calculate an intermediate-resolution 3D reconstruction of the authentic *Mr*NV virion, purified from infected *M*. *rosenbergii* post-larvae (a). This revealed a structure that, at this resolution, was indistinguishable from the VLP reconstruction. As in our VLP reconstruction, the virion map reveals the presence of an internal dodecahedral density that we attribute to viral genome (b). CryoEM, cryogenic electron microscopy; *Mr*NV, *M*. *rosenbergii* nodavirus; 3D, three-dimensional; VLP, virus-like particle.

To ensure that our atomic resolution model of the *Mr*NV capsid is an accurate description of the authentic virion, we determined the structure of purified virions at intermediate resolution. Virions were purified from homogenised, *Mr*NV-infected post-larvae and prepared for cryoEM. 3,931 particle images of frozen hydrated *Mr*NV virions were processed to produce a reconstruction at 6.6 Å resolution (Fig 7, S5 Fig). At this resolution, the map appears identical to that of the *Mr*NV VLP in all respects (compare Figs 7A and 1C). Furthermore, the packaged RNA shows a very similar, albeit noisier, structure to the previously described dodecahedral cage. Thus, we conclude that our high-resolution model is an accurate representation of the structure of the authentic *Mr*NV virion.

## Discussion

We have previously described the intermediate resolution structure of VLPs generated following recombinant expression of the *Mr*NV CP. This revealed a surprising divergence from known nodavirus structures [20]. The *Mr*NV T = 3 icosahedral capsid was seen to assemble with dimeric rather than the usual trimeric capsomeres. Consistent with previously published nodavirus structures, we found that the *Mr*NV VLP exhibited density suggestive of packaging of nucleic acids, most likely the cognate mRNA. We also observed a surprising difference in the orientations of the P-domain spikes between the 2 classes of dimer (AB and CC).

Here, we have extended this study, using cryoEM to calculate a 3D reconstruction of the *Mr*NV VLP at near-atomic resolution. This has allowed us to build an atomic model of the capsid’s asymmetric unit. Our model reveals that *Mr*NV, like most small icosahedral positive-sense RNA viruses, adopts the β-jelly–roll fold in the S domain. We identified major differences between AB and CC dimers in the linker region that connects the S and P domains, accounting for the radically different orientations of their respective P domains.

### Taxonomy of *Mr*NV

Beyond providing a detailed description of the structure of *Mr*NV, our data revealed startling similarities between the *Mr*NV capsid structure and those of tombusviruses. We found that the *Mr*NV capsid’s asymmetric unit is stabilised by 6 Ca^2+^ ions in a manner highly reminiscent of that seen in TBSV. Furthermore, the CP_C_ NTA was found to form an extensive network at the capsid interior that involved an interdigitated structure known as a β-annulus. This motif is also a feature of the tombusviruses. Finally, we found that the fold of the P domain consisted of a β-barrel that also bore a close resemblance to the P-domain structure of the tombusviruses.

**Fig 8:**
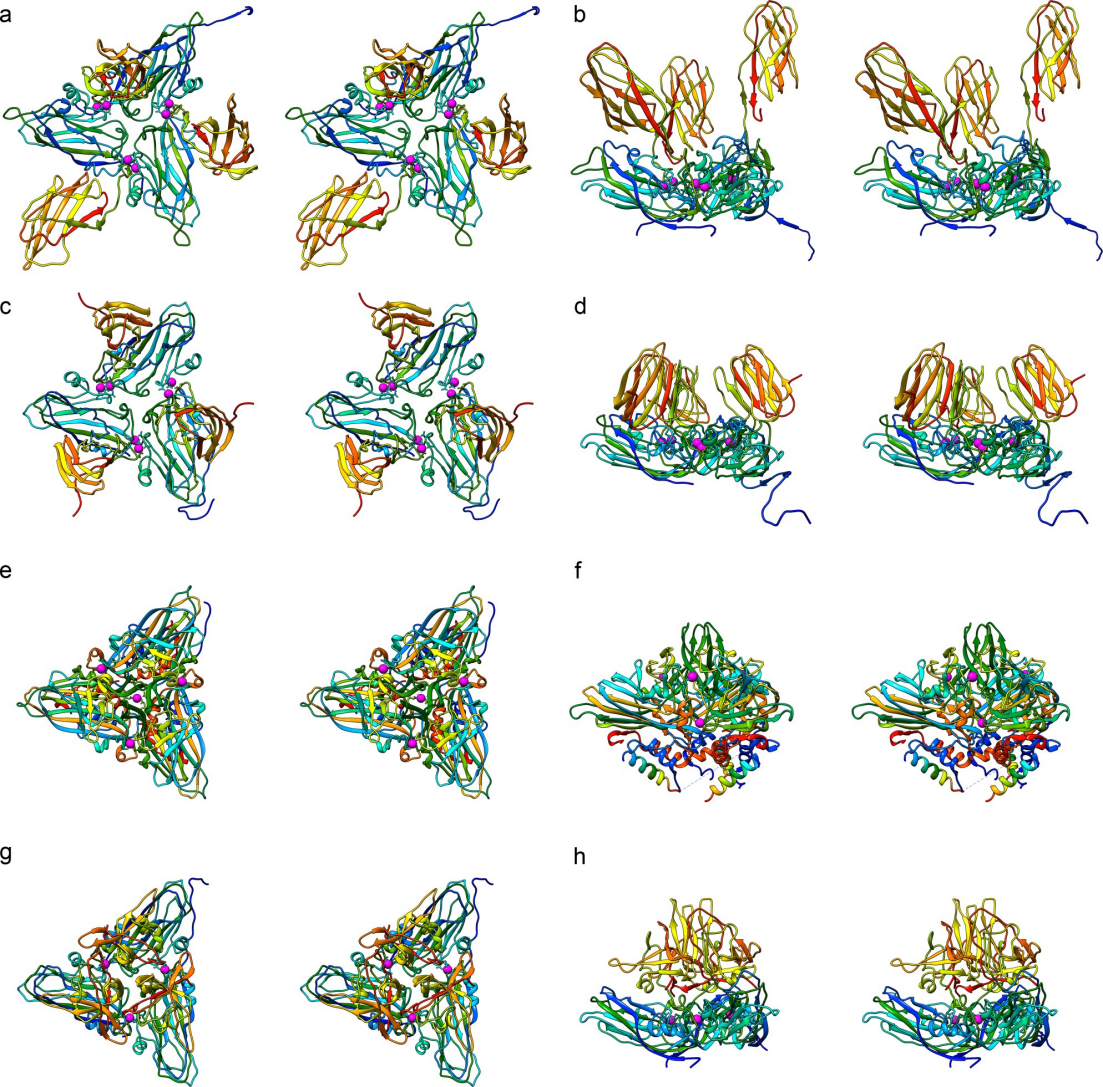
Divalent cation binding in nodavirus and tombusvirus capsid structures. Stereo-pair views of ribbon diagrams showing the asymmetric units for *Mr*NV (a,b), TBSV (PDB: 2TBV; c,d), FHV (PDB: 4FTB; e,f), and GNNV (PDB: 4WIZ; g,h). Views are presented along (left) and perpendicular to (right) the Q3 symmetry axis at the centre of the asymmetric unit. Ca^2+^ ions are coloured pink and represented with a larger radius to aid visualisation. FHV, Flock House virus; GNNV, grouper nervous necrosis virus; *Mr*NV, *M*. *rosenbergii* nodavirus; Q3, quasi-3–fold; PDB, Protein Data Bank; TBSV, tomato bushy stunt virus.

Capsid stabilisation by binding of divalent cations is well documented in both nodaviruses and tombusviruses (Fig 8). The alphanodavirus FHV and the betanodavirus GNNV have both been shown to bind a single Ca^2+^ ion at the interface between each CP subunit in the asymmetric unit (Fig 8E–8H) [17, 24]. FHV also binds a single Ca^2+^ ion at the quasi-3–fold (Q3) symmetry axis lying at the centre of the asymmetric unit (Fig 8E). Metal binding in *Mr*NV more closely resembles that seen in TBSV, however, in which 2 Ca^2+^ ions are bound by a DxDxxD motif [25].

There is no significant sequence homology between the CPs of alpha- and betanodaviruses, and comparison of known structures for these genera reveals substantial differences [17]. While both genera assemble capsids that have trimeric spikes, the FHV spike is small, comprising a single β-hairpin motif contributed by each CP subunit, whereas the GNNV CP has a distinct P domain that forms a more substantial capsomere. This feature was previously described as ‘TBSV like’ [26]. Alphanodavirus CPs fold such that both termini are located at the capsid interior; they also undergo proteolytic maturation to produce the γ-peptide, which is required for entry. Betanodaviruses and *Mr*NV both have their C-termini at the capsid exterior and do not encode a γ-peptide.

Like *Mr*NV, GNNV exhibits an extended CP_C_ NTA that crosses the icosahedral 2-fold symmetry axis [17]. The CP_C_ NTA also interacts with symmetry-related CP_C_ NTAs at the adjacent 3-fold axis and was described as forming a β-annulus; however, the CP_C_ NTA is not as extensive as that of *Mr*NV and importantly does not form the interdigitated β-strands that are characteristic of the β-annuli of the tombusviruses (and *Mr*NV). Rather, the network is stabilised by limited hydrogen bonding between CP_C_ NTAs at the icosahedral 3-fold axes.

Close comparison of the structures of the *Mr*NV capsid with those encoded by both genera of the Nodaviridae and by the Tombusviridae (Fig 8) led us to conclude that the structure of *Mr*NV more closely resembles that of the tombusviruses than either the alpha- or betanodaviruses. The divergence of amino acid sequence and distinct structural features of *Mr*NV compared with other nodaviruses supports the assertion that *Mr*NV, along with the related *Pv*NV, might be classified into a new genus, *Gammanodavirus* [10, 20]. Nodaviruses are characterised as small positive-sense RNA-containing viruses having bipartite genomes that infect fish and invertebrates. Tombusviruses are plant viruses that are classified on the nature of their RNA polymerases but are also seen to have consistent capsid structures. Thus, *Mr*NV having features of both virus families poses a conundrum with respect to its taxonomic status.

### P-domain structure and function

It has been demonstrated that the C-terminal domain of the *Mr*NV CP is important for virus attachment and entry. In particular, deletion of the last 26 amino acid residues substantially reduced infectivity. Inspection of the P-domain structure, however, strongly suggests that deletion of this region is liable to significantly disrupt the β-barrel, as it would remove 2 β-strands from the structure, one from the centre of each β-sheet (S6 Fig). Thus, while it seems likely that the receptor binding site is within the P domain, it may not be limited to the last 26 amino acid residues (345–371).

We have previously demonstrated insertion of heterologous epitopes into the *Mr*NV capsid structure at the C-ters, such as the ‘a’ determinant of hepatitis B virus surface antigen (HBsAg) [27] and the ectodomain of matrix 2 protein (M2e) of influenza A virus [28]. Both epitopes were shown to be displayed on the surface of VLPs. Thus, *Mr*NV VLPs present an attractive platform for antigen display. Our structure of *Mr*NV CP now allows us to refine the placement of foreign epitopes. Indeed, each of the 4 loops on the outer surface of the P domain (amino acid residues 268–275, 296–303, 322–326, and 350–355) represents potentially improved targets for further insertions. Combined with the capacity to package nucleic acids, *Mr*NV VLPS may therefore prove to be a useful tool for both vaccine and DNA/RNA delivery.

### An economically important pathogen of freshwater prawns

*Mr*NV threatens livelihoods and food security in developing nations. Our atomic-resolution model of the *Mr*NV capsid provides insights into the fundamental biology of this important pathogen, highlighting features that may prove important in our understanding of virus assembly or entry, such as the presence of metal ions that stabilise the asymmetric unit and the structure of the receptor-binding P domain. Such detailed understanding of the capsid structure provides a platform for the development of interventions to control or prevent disease outbreaks in the future.

## Materials and methods

### Construction of pGEM-T TARNA2 plasmid encoding *Mr*NV CP

The gene encoding *Mr*NV CP was amplified from plasmid pTrcHis2-TARNA2 [29]. The forward and reverse primers used to amplify the coding region were 5′-ATG GCC CTT AAC ATC ACC ATG GCT AGA GGT AAA CA-3′ (*Nco*I restriction site is underlined) and 5′-CTA TCG TCG GCA ATA ATT AAG GCG AAT TCG AAG CTT ACG T-3′ (*Eco*RI restriction site is underlined), respectively. PCR profile was denaturation at 95°C for 3 min, followed by 35 cycles of i) denaturation at 95°C for 30 s, ii) annealing at 59°C for 30 s, and iii) extension at 72°C for 1 min. The final extension was performed at 72°C for 10 min. The PCR products were excised and purified using the QIAquick Gel Extraction Kit (Qiagen, Hilden, Germany). The purified DNA was ligated with the linearised pGEM-T vector (Promega, Madison, WI, United States of America) and introduced into competent *Escherichia coli* DH5α cells. The transformants were plated on Luria Bertani (LB) agar plates containing ampicillin (100 μg/ml). Following an overnight incubation at 37°C, single bacterial colonies were picked and cultured in LB broth. The orientation and nucleotide sequence of the DNA insert were confirmed by DNA sequencing.

### Mutagenesis of pFastBac-HTC plasmid

To produce the *Mr*NV-CP without a His tag, the QuickChange II site-directed mutagenesis kit (Agilent Technologies, Santa Clara, CA, USA) was used to create an *Nco*I restriction-nuclease–cutting site in the pFastBac-HTC plasmid (Invitrogen, Carlsbad, CA, USA). The primers used for mutagenesis were 5′-CGG GCG CGG ATC TCG GTC CGA AAC CAT GGC GTA CTA CCA TCA CC-3′ and 5′-GGT GAT GGT AGT ACG CCA TGG TTT CGG ACC GAG ATC CGC GCC CG-3′, where the *Nco*I restriction-cutting site is underlined.

### Construction of recombinant bacmid pFastBac HT C-TARNA2

The pGEM-T TARNA2 plasmid and the mutated pFastBacHT-C plasmid were digested with *Eco*RI and *Nco*I, respectively. The digested products were purified using the QIAquick Gel Extraction Kit (Qiagen, Hilden, Germany) and ligated together to produce the pFastBacHTC-TARNA2. This was introduced into competent *E*. *coli* DH10Bac cells (Invitrogen, Carlsbad, CA, USA) and plated on LB agar plates containing kanamycin (50 μg/ml), gentamicin (7 μg/ml), and tetracycline (10 μg/ml). White bacterial colonies containing the recombinant plasmid were selected and cultured in LB broth. The recombinant bacmid DNA was extracted, and the presence of DNA insert was confirmed by PCR. The primers used in the PCR were pUC/M13 forward 5′-CCC AGT CAC GAC GTT GTA AAA CG-3′ and pUC/M13 reverse 5′-AGC GGA TAA CAA TTT CAC ACA GG-3′.

### Preparation of recombinant baculovirus stock

Sf9 cells (8 × 10^5^ cells/well) in a 6-well plate were transfected with the recombinant bacmid pFastBacHTC-TARNA2 using Cellfectin II reagent. The transfected cells were incubated at 27°C for 72 h. The cell culture medium was harvested by centrifugation at 500 × *g* for 5 min at 4°C. The P1 baculovirus stock was amplified by infecting the Sf9 cells (2 × 10^6^ cells/mL) in serum-free Sf-900 III SFM medium (Gibco, Gaithersburg, MD, USA). The infected cells were incubated at 27°C for 72 h. The P2 baculovirus stock was harvested by centrifugation at 500 × *g* for 5 min at 4°C and stored at 4°C for subsequent experiments.

### Expression and purification of recombinant *Mr*NV capsid

Sf9 cells were cultured as suspension cells at 27°C in a serum-free Sf-900 III SFM medium to reach a cell density of 2 × 10^6^ cells/ml. Recombinant baculovirus stock (10% [v/v]) was added into the culture, which was further incubated for 4 d at 27°C. The *Mr*NV capsid and the Sf9 cells were separated by centrifugation at 500 × *g* for 5 min at 4°C. The *Mr*NV capsid was precipitated at 60% (w/v) ammonium sulphate saturation for 2 h at 4°C. The proteins were pelleted by centrifugation at 18,000 × *g* for 20 min at 4°C. The pellet was resuspended in HEPES buffer A (20 mM HEPES, 100 mM NaCl; pH 7.4) and dialysed in the same buffer overnight. The dialysed sample was purified by size-exclusion chromatography (SEC) using a HiPrep 16/60 Sephacryl S-500 HR column (GE Healthcare, Chicago, IL, USA), which was attached to a fast protein liquid chromatography (FPLC) system (Akta Purifier; GE Healthcare, Chicago, IL, USA). The purified protein was concentrated with a 100 kDa molecular cutoff centrifugal concentrator (Pall, USA), and the protein concentration was determined with the Bradford assay [30].

### Isolation of authentic *Mr*NV virions from giant freshwater prawn larvae

Lysate of *Mr*NV-infected post-larvae was prepared according to published methods [31] with some modifications. Briefly, the infected post-larvae were homogenised in HEPES buffer B (25 mM HEPES, 150 mM NaCl; pH 7.4), and the homogenate was centrifuged at 6,000 × *g* for 10 min at 4°C to remove large debris. The supernatant was further clarified by centrifugation at 12,100 × *g* for 30 min at 4°C. The clarified supernatant was loaded onto a sucrose gradient (8–50% [w/v]) and centrifuged at 210,000 × *g* for 4.5 h at 4°C. Fractions (500 μl) were collected and analysed by SDS-PAGE and Western blotting. Fractions containing *Mr*NV were pooled and dialysed in HEPES buffer B. The purified *Mr*NV was concentrated by centrifugation using a centrifugal concentrator (molecular weight cutoff 10 kDa, Vivaspin Turbo 15, Sartorius, Göttingen, Germany). The final concentration of purified *Mr*NV was determined using the Bradford assay [30].

### CryoEM

Purified *Mr*NV VLPs (at approximately 0.2 mg/ml) or virions (at approximately 0.1 mg/ml) were prepared for cryogenic transmission electron microscopy using a Thermo-Fisher Vitrobot Mk IV (Thermo Fisher Scientific, Waltham, MA, USA). Particles were imaged on thin-continuous carbon films that had been applied to C-flat holey carbon support films (R1.2/1.3; Protochips, Morrisville, NC, USA). Four μl of VLP or virion preparation was loaded onto a grid for 1 min, blotted for 4 s, and plunged into liquid ethane. Vitrified samples were imaged at low-temperature (around 95 K) and under low-electron–dose conditions. To collect high-resolution data on *Mr*NV VLPs, grids were imaged at the eBIC, Diamond Light Source (UK) using a Thermo-Fisher Titan Krios (Thermo Fisher Scientific, Waltham, MA, USA) operated at 47,170× magnification. A total of 2,459 cryomicrograph movies were recorded on a Gatan K2 BioQuantum energy-filtered direct detector camera (Gatan, Pleasanton, CA, USA) operated in zero-loss imaging mode with a slit width of 20 eV. Five-s exposures were recorded in electron counting mode at a frame-rate of 4 frames per s and a dose rate of 1.8 electrons/pixel/frame. The pixel size was 1.06 Å/pixel. *Mr*NV virions were imaged using a JEOL 2200 FS cryo-microscope (JEOL, Tokyo, Japan) operated at a nominal magnification of 50,000× and an accelerating voltage of 200 kV. Frozen grids were held in a Gatan 626 cryo-stage (Gatan, Pleasanton, CA, USA). 263 cryomicrograph movies were recorded on a Direct Electron DE20 camera (San Diego, CA, USA) as 2-s exposures at 20 frames per s and approximately 1.5 electrons/pixel/frame. The pixel size was 1.11 Å/pixel.

### 3D image reconstruction

All image processing was performed using Relion 2.1 [32]. Image stacks of movie frames were motion-corrected using motioncor2 [33]. Defocus estimation was performed using GCTF [34]. For each dataset, a small subset of particle images was manually picked and subjected to 2D classification to prepare a template for automated particle picking. Thereafter, particles were automatically picked for all motion-corrected micrographs. Individual particle images were extracted in 512^2^-pixel boxes. For *Mr*NV VLPs, a total of 60,939 putative particles were extracted from motion-corrected micrographs and subjected to 2D classification. Class averages showing particle images with well-resolved structure were selected, reducing the dataset to 56,762 particles. 3D classification was then used to select the best particles for inclusion in the final reconstruction, further reducing the dataset to 40,883 particles. This dataset was then refined, leading to the calculation of a reconstruction with an overall resolution of 3.3 Å. The *Mr*NV virion reconstruction was calculated following an identical workflow in which 7,236 putative particle images were analysed, leading to the definition of a final dataset comprising 3,931 particle images. These data were reconstructed at a resolution of 6.6 Å. Reconstructions were evaluated to determine global and local resolution as well as estimated B-factors by postprocessing of maps calculated from randomised half sets of data, using the Relion postprocessing routine (S2 Table). In our study of *Mr*NV VLPs, we used our previously calculated 3D reconstruction as a template for starting the classification. For authentic virions, we performed the first 3D classification analysis using a Gaussian sphere as the starting model to prevent model bias, as previously described [20].

### Atomic model building

Atomic models were built from the high-resolution density maps using the CCP-EM suite of programmes [35], in particular COOT [36]. The model was refined using REFMAC [37] and PHENIX [38]. Validation was performed using MOLPROBITY [39]. Secondary structure assignment was performed using STRIDE (http://webclu.bio.wzw.tum.de/cgi-bin/stride/stridecgi.py) [40]. Density maps and atomic resolution models were visualised using UCSF Chimera [41]. Validation of metal ion assignment was performed using the ‘checkmymetal’ server (https://csgid.org/metal_sites) [42]. Contact interface analysis was performed using the PISA server (http://www.ebi.ac.uk/msd-srv/prot_int/cgi-bin/piserver) [43]. A 3D pairwise alignment of the *Mr*NV CP P-domain structure and that of CNV (PDB: 4LLF) was performed using the FatCat server (http://fatcat.burnham.org) [44]. Protein topology diagrams were generated using Pro-Origami (http://munk.csse.unimelb.edu.au/pro-origami/porun.shtml) [45] and edited using Inkscape (https://inkscape.org/en/).

### Data deposition

The cryoEM map of the *Mr*NV VLP was deposited in the Electron Microscopy Data Bank with accession number EMD-0129. The cryoEM map of the *Mr*NV virion was deposited in the Electron Microscopy Data Bank with accession number EMD-0130. The atomic coordinates for the asymmetric unit of the *Mr*NV VLP were deposited in the PDB with accession number PDB: 6H2B. The cryoEM image data for EMD-0129 are deposited in EMPIAR as motion-corrected single-frame micrographs with accession number EMPIAR-10203.

## Supporting information

S1 Fig FSC plot for the *Mr*NV VLP 3D reconstruction (blue), showing that the overall resolution of the map was 3.3 Å. The FSC validation curve (phase randomised from 4.5 Å) is shown (red). FSC, Fourier Shell Correlation; *Mr*NV, *M*. *rosenbergii* nodavirus; 3D, three-dimensional; VLP, virus-like particle.
(TIF)

S2 Fig The full-length sequence of the *Mr*NV CP. CP, capsid protein; *Mr*NV, *M*. *rosenbergii* nodavirus.
(PDF)

S3 Fig Stereo pair views of the density map for the AB-dimer spike (transparent isosurface). A ribbon diagram is shown for CPB highlighting the protein topology for this domain. Two views are presented, rotated 180° about the y-axis (a,b). CP, capsid protein.
(TIF)

S4 Fig Pairwise 3D alignment of the CP_B_ P domains of cucumber mosaic virus (PDB: 4LLF) and *Mr*NV. β-sheets are indicated as blue arrows. CP, capsid protein; *Mr*NV, *M*. *rosenbergii* nodavirus; P domain, protruding domain; PDB, Protein Data Bank; 3D, three-dimensional.
(PDF)

S5 Fig FSC plot for the *Mr*NV virion 3D reconstruction (blue), showing that the overall resolution of the map was 6.6 Å. The FSC validation curve (phase randomised from 9.6 Å) is shown (red). FSC, Fourier Shell Correlation; *Mr*NV, *M*. *rosenbergii* nodavirus; 3D, three-dimensional.
(TIF)

S6 Fig The AB P-domain structure, coloured to show the amino acid residues 345–371, identified as a putative receptor binding site (blue). P domain, protruding domain.
(TIF)

S1 Movie Movie to show the icosahedral reconstruction of the *Mr*NV VLP. *Mr*NV, *M*. *rosenbergii* nodavirus; VLP, virus-like particle.
(MP4)

S2 Movie Movie to show the atomic model of the *Mr*NV CP. CP, capsid protein; *Mr*NV, *M*. *rosenbergii* nodavirus.
(MP4)

S3 Movie Movie to show detailed features of the CP_C_ NTA and β-annulus structure. CP, capsid protein, NTA N-terminal arm.
(MP4)

S4 Movie Movie to show the position of metal-binding sites in the *Mr*NV asymmetric unit. *Mr*NV, *M*. *rosenbergii* nodavirus.
(MP4)

S5 Movie Movie to show how 2 AB spikes and 1 CC spike create a superstructure across each icosahedral 2-fold axis of the *Mr*NV capsid. *Mr*NV, *M*. *rosenbergii* nodavirus.
(MP4)

S1 Table Molprobity validation of the *Mr*NV capsid model. *Mr*NV, *M*. *rosenbergii* nodavirus.
(PDF)

S2 Table Image processing statistics for the cryoEM reconstructions of the *Mr*NV VLP and virion. CryoEM, cryogenic electron microscopy; *Mr*NV, *M*. *rosenbergii* nodavirus; VLP, virus-like particle.
(PDF)

## Abbreviations

BBV: black beetle virus
BoV: Boolarra virus
CNV: cucumber necrosis virus
CP: capsid protein
cryoEM: cryogenic electron microscopy
C-ter: C-terminus
eBIC: Electron Bio-Imaging Centre
FHV: Flock House virus
FPLC: fast protein liquid chromatography
GNNV: grouper nervous necrosis virus
HBsAg: hepatitis B virus surface antigen
LB: Luria Bertani
M2e: ectodomain of matrix 2 protein
MGNNV: Malabaricus grouper nervous necrosis virus
*Mr*NV: *Macrobrachium rosenbergii* nodavirus
NLS: nuclear localisation signal
NoV: Nodamura virus
NTA: N-terminal arm
N-ter: N-terminus
P domain: protruding domain
PaV: Pariacoto virus
PDB: Protein Data Bank
*Pv*NV: *Penaeus vannamei* nodavirus
Q3: quasi-3–fold
RdRP: RNA-dependent RNA polymerase
S domain: shell domain
SEC: size-exclusion chromatography
Sf9: *Spodoptera frugiperda*
SJNNV: striped jack nervous necrosis virus
3D: three-dimensional
TBSV: tomato bushy stunt virus
VLP: virus-like particle
WTD: white-tail disease

## References

[1] FAO. Macrobrachium rosenbergii (De Man, 1879): Fishers and Aquaculture Department, Food and Agricultural Organization of the United Nations; 2017 [cited 2018 August 3]. Available from: http://www.fao.org/fishery/culturedspecies/Macrobrachium_rosenbergii/en.

[2] Sahul Hameed AS, Bonami JR. White Tail Disease of Freshwater Prawn, Macrobrachium rosenbergii. Indian J Virol. 2012;23(2):134–40. 10.1007/s13337-012-0087-y ; PubMed Central PMCID: PMCPMC3550746. 23997437 PMC3550746

[3] Qian D, Shi Z, Zhang S, Cao Z, Liu W, Li L, et al Extra small virus-like particles (XSV) and nodavirus associated with whitish muscle disease in the giant freshwater prawn, Macrobrachium rosenbergii. J Fish Dis. 2003;26(9):521–7. 10.1046/j.1365-2761.2003.00486.x . 14575370

[4] Hameed ASS, Yoganandhan K, Widada JS, Bonami JR. Experimental transmission and tissue tropism of Macrobrachium rosenbergii nodavirus (MrNV) and its associated extra small virus (XSV). Diseases of Aquatic Organisms. 2004;62(3):191–6. 10.3354/dao062191 15672874

[5] Hsieh CY, Wu ZB, Tung MC, Tu C, Lo SP, Chang TC, et al In situ hybridization and RT-PCR detection of Macrobrachium rosenbergii nodavirus in giant freshwater prawn, Macrobrachium rosenbergii (de Man), in Taiwan. J Fish Dis. 2006;29(11):665–71. 10.1111/j.1365-2761.2006.00762.x . 17169113

[6] Yoganandhan K, Manee L, Supamas S, Chalor L. White tail disease of the giant freshwater prawn Macrobrachium rosenbergii in Thailand. Diseases of Aquatic Organisms. 2006;69(2–3):255–8. 10.3354/dao069255 16724570

[7] Saedi TA, Moeini H, Tan WS, Yusoff K, Daud HM, Chu KB, et al Detection and phylogenetic profiling of nodavirus associated with white tail disease in Malaysian Macrobrachium rosenbergii de Man. Mol Biol Rep. 2012;39(5):5785–90. 10.1007/s11033-011-1389-7 . 22223294

[8] Hayakijkosol O, Owens L. Non-permissive C6/36 cell culture for the Australian isolate of Macrobrachium rosenbergii nodavirus. J Fish Dis. 2013;36(4):401–9. 10.1111/j.1365-2761.2012.01414.x . 23134578

[9] Murwantoko M, Bimantara A, Roosmanto R, Kawaichi M. Macrobrachium rosenbergii nodavirus infection in a giant freshwater prawn hatchery in Indonesia. Springerplus. 2016;5(1):1729 10.1186/s40064-016-3127-z ; PubMed Central PMCID: PMCPMC5053962. 27777864 PMC5053962

[10] NaveenKumar S, Shekar M, Karunasagar I, Karunasagar I. Genetic analysis of RNA1 and RNA2 of Macrobrachium rosenbergii nodavirus (MrNV) isolated from India. Virus Res. 2013;173(2):377–85. 10.1016/j.virusres.2013.01.003 . 23318596

[11] Goh ZH, Mohd NA, Tan SG, Bhassu S, Tan WS. RNA-binding region of Macrobrachium rosenbergii nodavirus capsid protein. J Gen Virol. 2014;95(Pt 9):1919–28. 10.1099/vir.0.064014-0 . 24878641

[12] Somrit M, Watthammawut A, Chotwiwatthanakun C, Ounjai P, Suntimanawong W, Weerachatyanukul W. C-terminal domain on the outer surface of the Macrobrachium rosenbergii nodavirus capsid is required for Sf9 cell binding and internalization. Virus Res. 2016;227:41–8. 10.1016/j.virusres.2016.09.017 . 27693291

[13] Hanapi UF, Yong CY, Goh ZH, Alitheen NB, Yeap SK, Tan WS. Tracking the virus-like particles of Macrobrachium rosenbergii nodavirus in insect cells. PeerJ. 2017;5:e2947 10.7717/peerj.2947 ; PubMed Central PMCID: PMCPMC5301976. 28194311 PMC5301976

[14] Fisher AJ, Johnson JE. Ordered duplex RNA controls capsid architecture in an icosahedral animal virus. Nature. 1993;361(6408):176–9. 10.1038/361176a0 . 8421524

[15] Tang L, Johnson KN, Ball LA, Lin T, Yeager M, Johnson JE. The structure of pariacoto virus reveals a dodecahedral cage of duplex RNA. Nat Struct Biol. 2001;8(1):77–83. 10.1038/83089 . 11135676

[16] Wery JP, Reddy VS, Hosur MV, Johnson JE. The refined three-dimensional structure of an insect virus at 2.8 A resolution. J Mol Biol. 1994;235(2):565–86. 10.1006/jmbi.1994.1014 . 8289282

[17] Chen NC, Yoshimura M, Guan HH, Wang TY, Misumi Y, Lin CC, et al Crystal Structures of a Piscine Betanodavirus: Mechanisms of Capsid Assembly and Viral Infection. PLoS Pathog. 2015;11(10):e1005203 10.1371/journal.ppat.1005203 ; PubMed Central PMCID: PMCPMC4619592. 26491970 PMC4619592

[18] Zlotnick A, Natarajan P, Munshi S, Johnson JE. Resolution of space-group ambiguity and structure determination of nodamura virus to 3.3 A resolution from pseudo-R32 (monoclinic) crystals. Acta Crystallogr D Biol Crystallogr. 1997;53(Pt 6):738–46. 10.1107/S0907444997007427 . 15299863

[19] Kueh CL, Yong CY, Masoomi Dezfooli S, Bhassu S, Tan SG, Tan WS. Virus-like particle of Macrobrachium rosenbergii nodavirus produced in Spodoptera frugiperda (Sf9) cells is distinctive from that produced in Escherichia coli. Biotechnol Prog. 2017;33(2):549–557. Epub 2016 Nov 29. 10.1002/btpr.2409 . 27860432

[20] Ho KL, Kueh CL, Beh PL, Tan WS, Bhella D. Cryo-Electron Microscopy Structure of the Macrobrachium rosenbergii Nodavirus Capsid at 7 Angstroms Resolution. Sci Rep. 2017;7(1):2083 Epub 2017/05/20. 10.1038/s41598-017-02292-0 ; PubMed Central PMCID: PMCPMC5437026. 28522842 PMC5437026

[21] Harrison SC, Olson AJ, Schutt CE, Winkler FK, Bricogne G. Tomato bushy stunt virus at 2.9 A resolution. Nature. 1978;276(5686):368–73. Epub 1978/11/23. . 19711552 10.1038/276368a0

[22] Hogle J, Kirchhausen T, Harrison SC. Divalent cation sites in tomato bushy stunt virus. Difference maps at 2–9 A resolution. J Mol Biol. 1983;171(1):95–100. Epub 1983/11/25. . 6417343 10.1016/s0022-2836(83)80315-5

[23] Li M, Kakani K, Katpally U, Johnson S, Rochon D, Smith TJ. Atomic structure of Cucumber necrosis virus and the role of the capsid in vector transmission. J Virol. 2013;87(22):12166–75. doi: 10.1128/JVI.01965-13 PubMed PMID: 24006433; PubMed Central PMCID: PMCPMC3807921. 24006433 PMC3807921

[24] Banerjee M, Speir JA, Kwan MH, Huang R, Aryanpur PP, Bothner B, et al Structure and function of a genetically engineered mimic of a nonenveloped virus entry intermediate. J Virol. 2010;84(9):4737–46. Epub 2010/02/19. 10.1128/JVI.02670-09 ; PubMed Central PMCID: PMCPMC2863772. 20164221 PMC2863772

[25] Hopper P, Harrison SC, Sauer RT. Structure of tomato bushy stunt virus. V. Coat protein sequence determination and its structural implications. J Mol Biol. 1984;177(4):701–13. Epub 1984/08/25. . 6481803 10.1016/0022-2836(84)90045-7

[26] Tang L, Lin CS, Krishna NK, Yeager M, Schneemann A, Johnson JE. Virus-like particles of a fish nodavirus display a capsid subunit domain organization different from that of insect nodaviruses. J Virol. 2002;76(12):6370–5. 10.1128/JVI.76.12.6370-6375.2002 ; PubMed Central PMCID: PMCPMC136213. 12021370 PMC136213

[27] Yong CY, Yeap SK, Goh ZH, Ho KL, Omar AR, Tan WS. Induction of humoral and cell-mediated immune responses by hepatitis B virus epitope displayed on the virus-like particles of prawn nodavirus. Appl Environ Microbiol. 2015;81(3):882–9. 10.1128/AEM.03695-14 ; PubMed Central PMCID: PMCPMC4292494. 25416760 PMC4292494

[28] Yong CY, Yeap SK, Ho KL, Omar AR, Tan WS. Potential recombinant vaccine against influenza A virus based on M2e displayed on nodaviral capsid nanoparticles. Int J Nanomedicine. 2015;10:2751–63. 10.2147/IJN.S77405 ; PubMed Central PMCID: PMCPMC4396508. 25897220 PMC4396508

[29] Goh ZH, Tan SG, Bhassu S, Tan WS. Virus-like particles of Macrobrachium rosenbergii nodavirus produced in bacteria. J Virol Methods. 2011;175(1):74–9. 10.1016/j.jviromet.2011.04.021 . 21536072

[30] Bradford MM. A rapid and sensitive method for the quantitation of microgram quantities of protein utilizing the principle of protein-dye binding. Anal Biochem. 1976;72:248–54. Epub 1976/05/07. . 942051 10.1016/0003-2697(76)90527-3

[31] Somrit M, Watthammawut A, Chotwiwatthanakun C, Weerachatyanukul W. The key molecular events during Macrobrachium rosenbergii nodavirus (MrNV) infection and replication in Sf9 insect cells. Virus Res. 2016;223:1–9. 10.1016/j.virusres.2016.06.012 . 27327530 PMC7126520

[32] Scheres SH. RELION: implementation of a Bayesian approach to cryo-EM structure determination. J Struct Biol. 2012;180(3):519–30. 10.1016/j.jsb.2012.09.006 ; PubMed Central PMCID: PMCPMC3690530. 23000701 PMC3690530

[33] Zheng SQ, Palovcak E, Armache JP, Verba KA, Cheng YF, Agard DA. MotionCor2: anisotropic correction of beam-induced motion for improved cryo-electron microscopy. Nature Methods. 2017;14(4):331–2. WOS:000397900500004. 10.1038/nmeth.4193 28250466 PMC5494038

[34] Zhang K. Gctf: Real-time CTF determination and correction. J Struct Biol. 2016;193(1):1–12. 10.1016/j.jsb.2015.11.003 ; PubMed Central PMCID: PMCPMC4711343. 26592709 PMC4711343

[35] Burnley T, Palmer CM, Winn M. Recent developments in the CCP-EM software suite. Acta Crystallogr D Struct Biol. 2017;73(Pt 6):469–77. Epub 2017/06/06. 10.1107/S2059798317007859 ; PubMed Central PMCID: PMCPMC5458488. 28580908 PMC5458488

[36] Emsley P, Cowtan K. Coot: model-building tools for molecular graphics. Acta Crystallogr D Biol Crystallogr. 2004;60(Pt 12 Pt 1):2126–32. Epub 2004/12/02. 10.1107/S0907444904019158 . 15572765

[37] Murshudov GN, Vagin AA, Dodson EJ. Refinement of macromolecular structures by the maximum-likelihood method. Acta Crystallogr D Biol Crystallogr. 1997;53(Pt 3):240–55. Epub 1997/05/01. 10.1107/S0907444996012255 . 15299926

[38] Adams PD, Afonine PV, Bunkoczi G, Chen VB, Davis IW, Echols N, et al PHENIX: a comprehensive Python-based system for macromolecular structure solution. Acta Crystallogr D Biol Crystallogr. 2010;66(Pt 2):213–21. Epub 2010/02/04. 10.1107/S0907444909052925 ; PubMed Central PMCID: PMCPMC2815670. 20124702 PMC2815670

[39] Chen VB, Arendall WB, 3rd, Headd JJ, Keedy DA, Immormino RM, Kapral GJ, et al MolProbity: all-atom structure validation for macromolecular crystallography. Acta Crystallogr D Biol Crystallogr. 2010;66(Pt 1):12–21. Epub 2010/01/09. 10.1107/S0907444909042073 ; PubMed Central PMCID: PMCPMC2803126. 20057044 PMC2803126

[40] Frishman D, Argos P. Knowledge-based protein secondary structure assignment. Proteins. 1995;23(4):566–79. Epub 1995/12/01. 10.1002/prot.340230412 . 8749853

[41] Pettersen EF, Goddard TD, Huang CC, Couch GS, Greenblatt DM, Meng EC, et al UCSF Chimera—a visualization system for exploratory research and analysis. J Comput Chem. 2004;25(13):1605–12. 10.1002/jcc.20084 . 15264254

[42] Zheng H, Cooper DR, Porebski PJ, Shabalin IG, Handing KB, Minor W. CheckMyMetal: a macromolecular metal-binding validation tool. Acta Crystallogr D Struct Biol. 2017;73(Pt 3):223–33. Epub 2017/03/16. 10.1107/S2059798317001061 ; PubMed Central PMCID: PMCPMC5349434. 28291757 PMC5349434

[43] Krissinel E, Henrick K. Inference of macromolecular assemblies from crystalline state. J Mol Biol. 2007;372(3):774–97. Epub 2007/08/08. 10.1016/j.jmb.2007.05.022 . 17681537

[44] Ye Y, Godzik A. FATCAT: a web server for flexible structure comparison and structure similarity searching. Nucleic Acids Res. 2004;32(Web Server issue):W582–5. Epub 2004/06/25. 10.1093/nar/gkh430 ; PubMed Central PMCID: PMCPMC441568. 15215455 PMC441568

[45] Stivala A, Wybrow M, Wirth A, Whisstock JC, Stuckey PJ. Automatic generation of protein structure cartoons with Pro-origami. Bioinformatics. 2011;27(23):3315–6. Epub 2011/10/14. 10.1093/bioinformatics/btr575 . 21994221

