# Supplementary figures and images for: Structure of the Macrobrachium rosenbergii nodavirus: A new genus within the Nodaviridae?
Source: PLoS Biol. 2018 Oct 22;16(10):e3000038. doi: 10.1371/journal.pbio.3000038 (PMC6211762; doi:10.1371/journal.pbio.3000038)

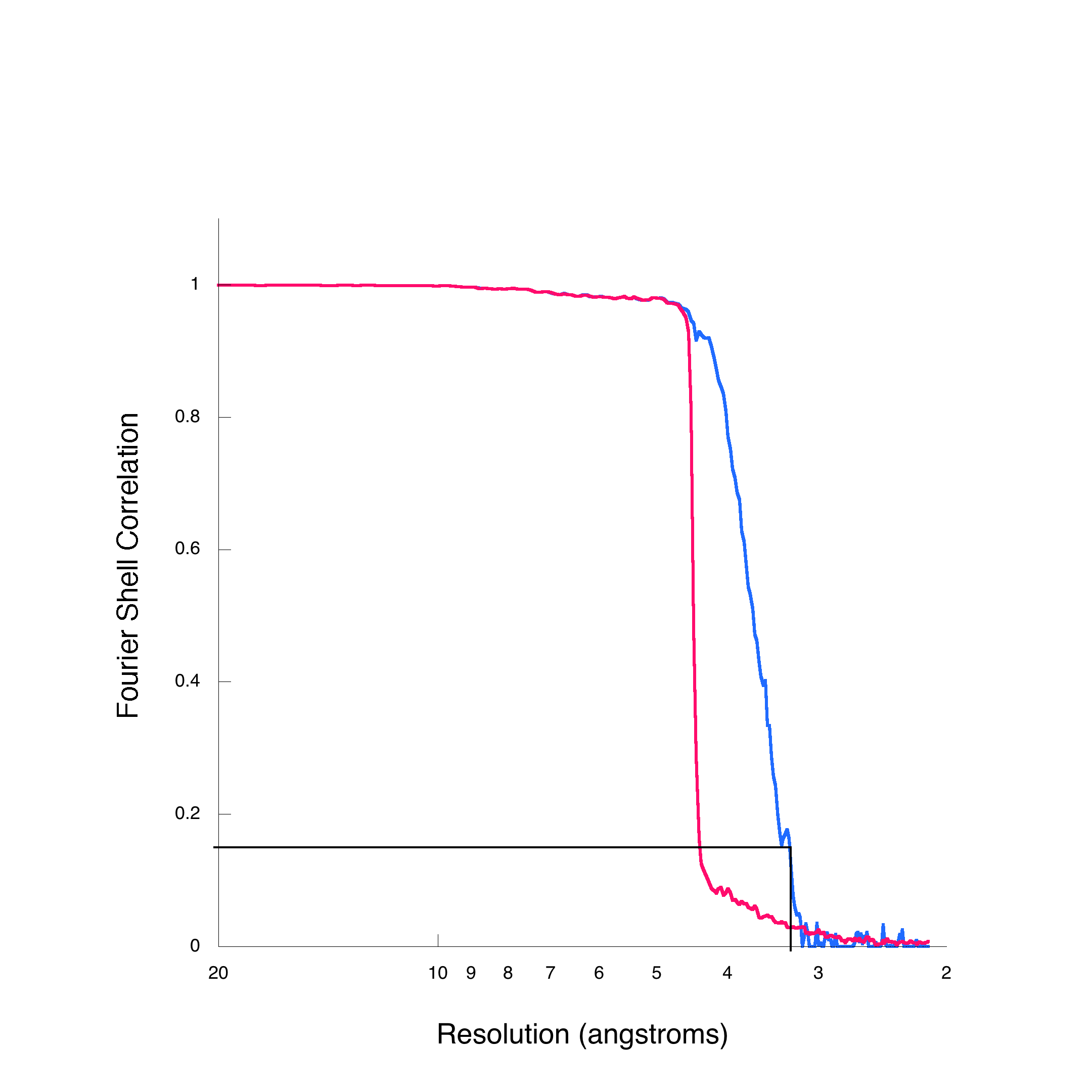

Supplement: S1 Fig — The FSC validation curve (phase randomised from 4.5 Å) is shown (red). FSC, Fourier Shell Correlation; MrNV, M. rosenbergii nodavirus; 3D, three-dimensional; VLP, virus-like particle. (TIF) [file pbio.3000038.s001.tif]

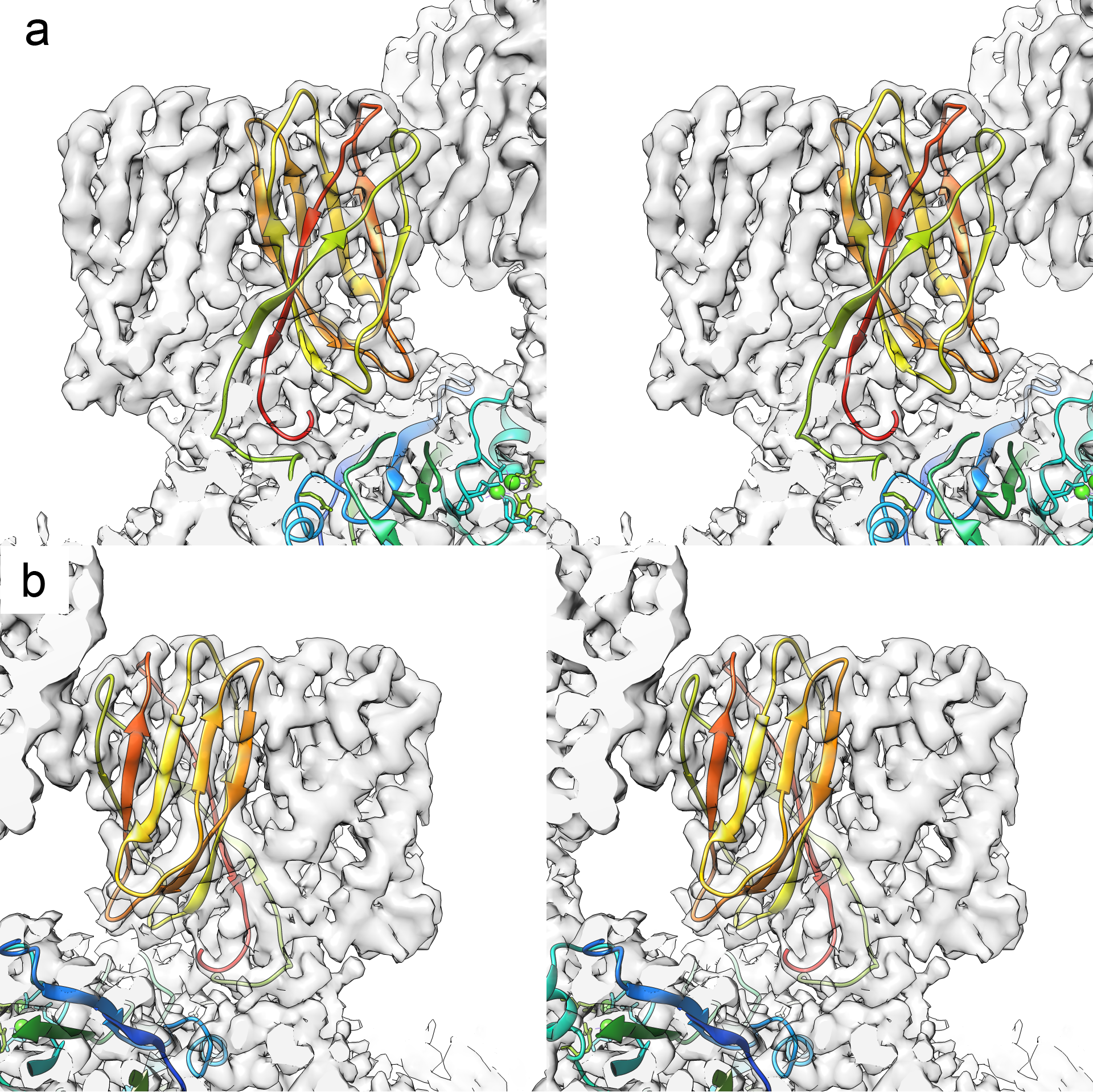

Supplement: S3 Fig — A ribbon diagram is shown for CPB highlighting the protein topology for this domain. Two views are presented, rotated 180° about the y-axis (a,b). CP, capsid protein. (TIF) [file pbio.3000038.s003.tif]

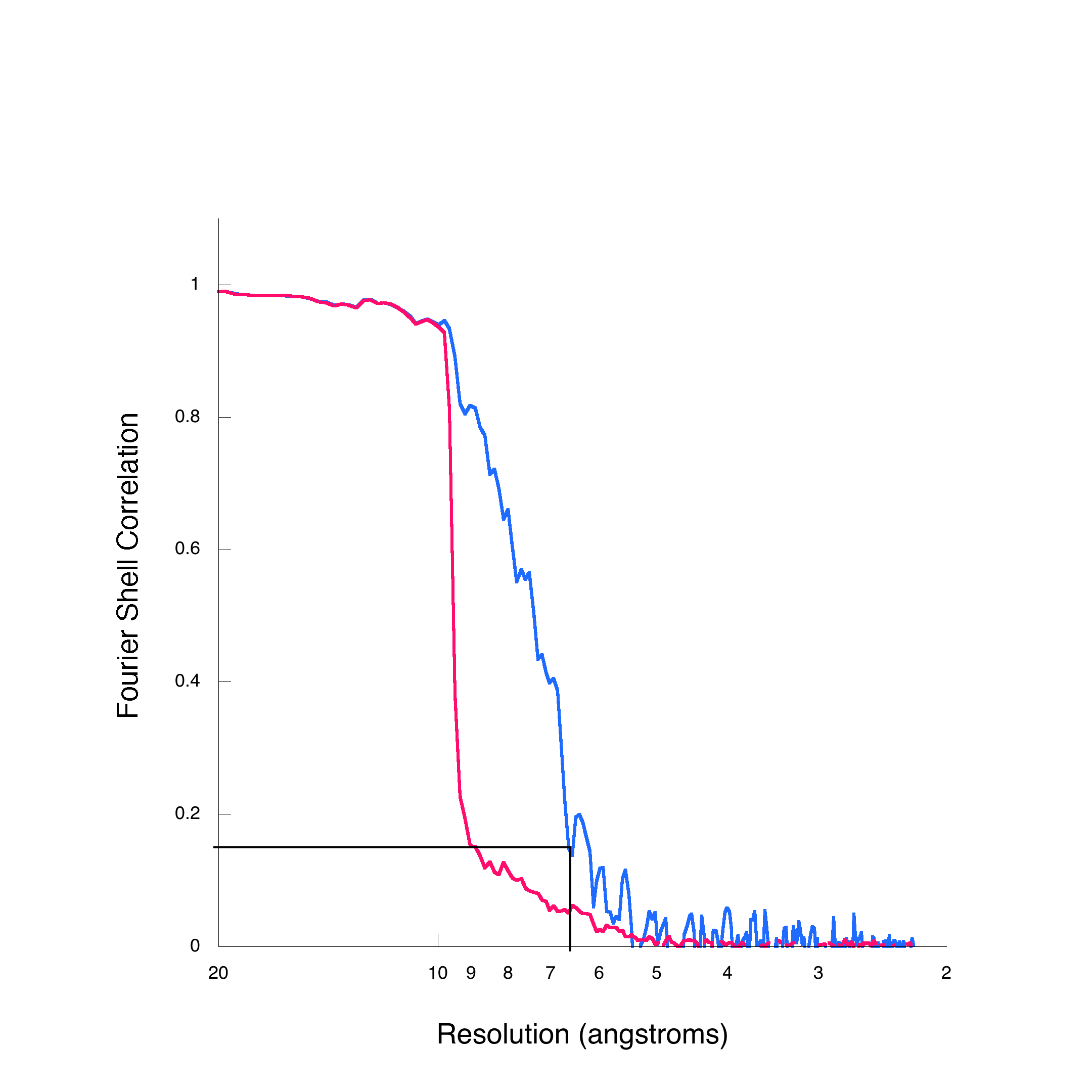

Supplement: S5 Fig — The FSC validation curve (phase randomised from 9.6 Å) is shown (red). FSC, Fourier Shell Correlation; MrNV, M. rosenbergii nodavirus; 3D, three-dimensional. (TIF) [file pbio.3000038.s005.tif]

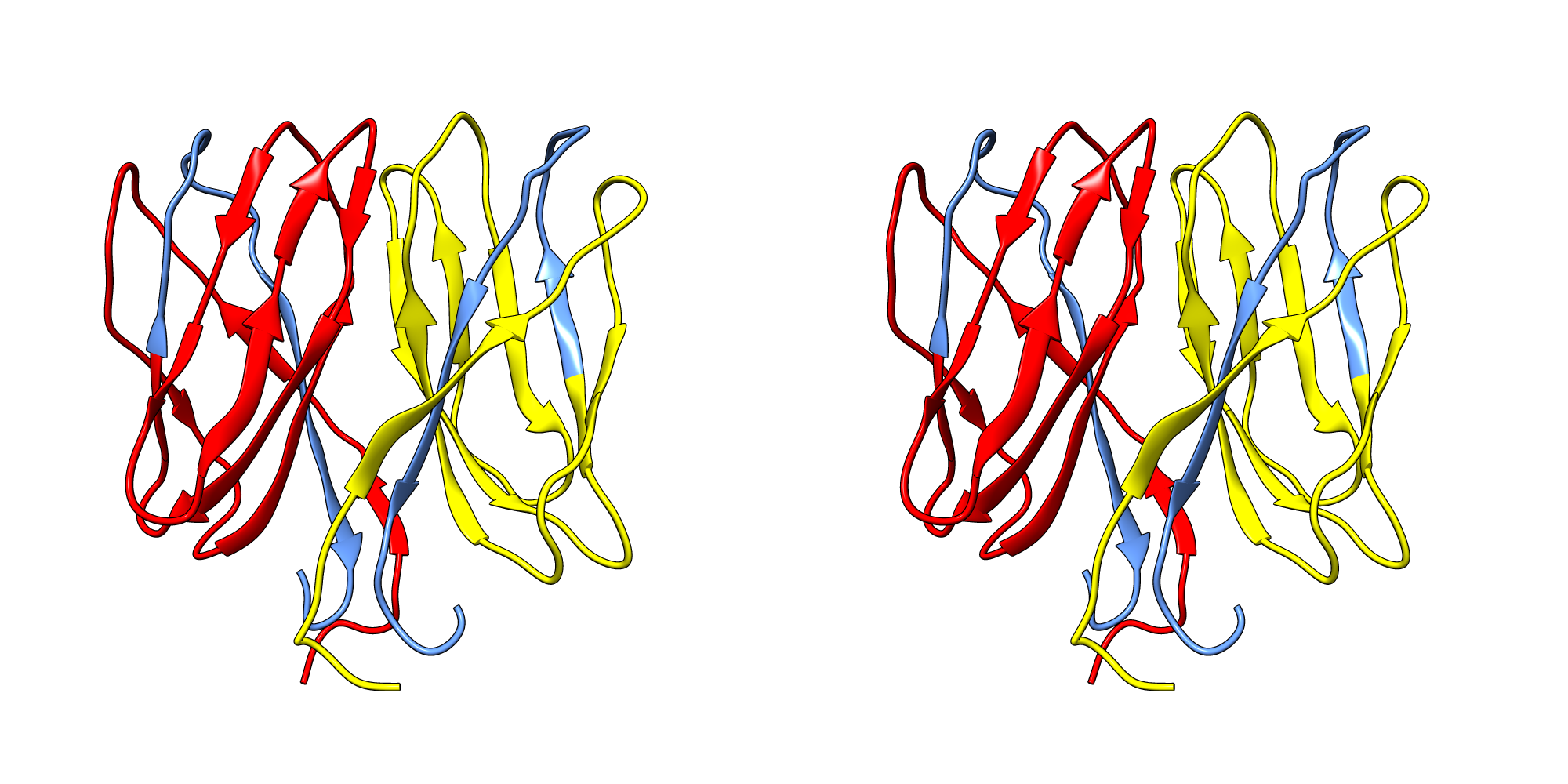

Supplement: S6 Fig — P domain, protruding domain. (TIF) [file pbio.3000038.s006.tif]
