# Supplementary material for: Structure of the Macrobrachium rosenbergii nodavirus: A new genus within the Nodaviridae?
Source: PLoS Biol. 2018 Oct 22;16(10):e3000038. doi: 10.1371/journal.pbio.3000038 (PMC6211762; doi:10.1371/journal.pbio.3000038)
Supplement: S2 Fig — CP, capsid protein; MrNV, M. rosenbergii nodavirus. (PDF) [file pbio.3000038.s002.pdf]

```
  1 MARGKQNSNQ AQNNSNANGK RRKRSRRNRN PQTIPNFNPI VAKPTVAPLQ TNIRSARSDV
 61 NAITVLNGSD FLTTVKVRGS NNLTDSKSRI LVKQPISASS FLGTRISGLS QFWERYRWHK
121 AAVRYVPAVP NTLACQLIGY IDTDPLDDPN VILDVDQLLR QATSQVGARQ WNFSDTTTIP
181 LIVRRDDQLY YTGQDKENVR FSQQGVFYLL QVTTLLNISG EAITNDLISG SLYLDWVCGF
241 SMPQINPTPV EISQLTYNAD TIGNWVPPTE LNQTYTQDIT GLKPNSKFII VPYMDRTSSE
301 VLQKCTITCN EVNAVGSISY FDTNDIKCNG YITFQANNIG EATFTLVTDY KGVTDAKPYQ
361 YRIIRAIVGN N
```
