# Supplementary material for: Structure of the Macrobrachium rosenbergii nodavirus: A new genus within the Nodaviridae?
Source: PLoS Biol. 2018 Oct 22;16(10):e3000038. doi: 10.1371/journal.pbio.3000038 (PMC6211762; doi:10.1371/journal.pbio.3000038)
Supplement: S4 Fig — β-sheets are indicated as blue arrows. CP, capsid protein; MrNV, M. rosenbergii nodavirus; P domain, protruding domain; PDB, Protein Data Bank; 3D, three-dimensional. (PDF) [file pbio.3000038.s004.pdf]

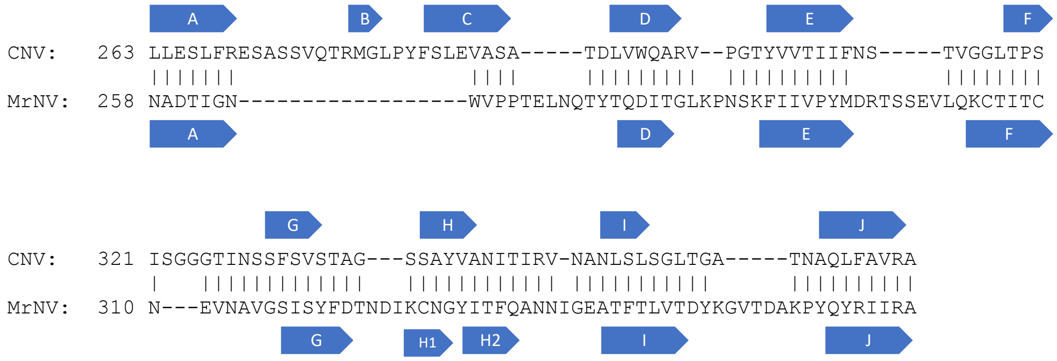
A B C D E F
CNV: 263 LLESLFRESASSVQTRMGLPYFSLEVASA-----TDLVWQARV--PGTYVVTIIFNS-----TVGGLTPS
MrNV: 258 NADTIGN------------------WVPPTELNQTYTQDITGLKPNSKFIIVPYMDRTSSEVLQKCTITC
A D E F
G H I J
CNV: 321 ISGGGTINSSFSVSTAG---SSAYVANITIRV-NANLSLSGLTGA-----TNAQLFAVRA
MrNV: 310 N---EVNAVGSISYFDTNDIKCNGYITFQANNIGEATFTLVTDYKGVTDAKPYQYRIIRA
G H1 H2 I J
