# Supplementary material for: Structure of the Macrobrachium rosenbergii nodavirus: A new genus within the Nodaviridae?
Source: PLoS Biol. 2018 Oct 22;16(10):e3000038. doi: 10.1371/journal.pbio.3000038 (PMC6211762; doi:10.1371/journal.pbio.3000038)
Supplement: S1 Table — MrNV, M. rosenbergii nodavirus. (PDF) [file pbio.3000038.s012.pdf]

Model to map fit - CC (around atoms)	0.779
Ramachandran outliers	0.10 %
Ramachandran favoured	87.09 %
Rotamer outliers	0.00 %
C-beta deviations	0
Clashscore	4.66
RMS (bonds)	0.0111
RMS (angles)	1.25
MolProbity score	1.86
