# Supplementary material for: Structure of the Macrobrachium rosenbergii nodavirus: A new genus within the Nodaviridae?
Source: PLoS Biol. 2018 Oct 22;16(10):e3000038. doi: 10.1371/journal.pbio.3000038 (PMC6211762; doi:10.1371/journal.pbio.3000038)
Supplement: S2 Table — CryoEM, cryogenic electron microscopy; MrNV, M. rosenbergii nodavirus; VLP, virus-like particle. (PDF) [file pbio.3000038.s013.pdf]

Reconstruction	*Mr*NV VLP
Number of Particles	40,883
Gold-Standard Resolution (FSC 0.143)	3.3 Å
B-factor	-133 $Å^2$
Reconstruction	*Mr*NV Virion
Number of Particles	3,931
Gold-Standard Resolution (FSC 0.143)	6.6 Å
B-factor	-413 $Å^2$
